# Perpetuation of Avian Influenza from Molt to Fall Migration in Wild Swan Geese (*Anser cygnoides*): An Agent-Based Modeling Approach

**DOI:** 10.3390/v17020196

**Published:** 2025-01-30

**Authors:** John Y. Takekawa, Chang-Yong Choi, Diann J. Prosser, Jeffery D. Sullivan, Nyambayar Batbayar, Xiangming Xiao

**Affiliations:** 1 U.S. Geological Survey, Western Ecological Research Center, Vallejo, CA 94592, USA; 2 School of Biological Sciences, University of Oklahoma, Norman, OK 73019, USA; xiangming.xiao@ou.edu; 3 Department of Forest Sciences, Seoul National University, Seoul 08826, Republic of Korea; 4 U.S. Geological Survey, Eastern Ecological Science Center, Laurel, MD 20708, USA; dprosser@usgs.gov (D.J.P.); jdsullivan@usgs.gov (J.D.S.); 5 Wildlife Science and Conservation Center, Ulaanbaatar 210351, Mongolia

**Keywords:** highly pathogenic avian influenza, HPAI, movement ecology, agent-based model, SEIR model, susceptible–exposed–infectious–recovered, swan goose, telemetry

## Abstract

Wild waterfowl are considered to be the reservoir of avian influenza, but their distinct annual life cycle stages and their contribution to disease dynamics are not well understood. Studies of the highly pathogenic avian influenza (HPAI) virus have primarily focused on wintering grounds, where human and poultry densities are high year-round, compared with breeding grounds, where migratory waterfowl are more isolated. Few if any studies of avian influenza have focused on the molting stage where wild waterfowl congregate in a few selected wetlands and undergo the simultaneous molt of wing and tail feathers during a vulnerable flightless period. The molting stage may be one of the most important periods for the perpetuation of the disease in waterfowl, since during this stage, immunologically naïve young birds and adults freely intermix prior to the fall migration. Our study incorporated empirical data from virological field samplings and markings of Swan Geese (*Anser cygnoides*) on their breeding grounds in Mongolia in an integrated agent-based model (ABM) that included susceptible–exposed–infectious–recovered (SEIR) states. Our ABM results provided unique insights and indicated that individual movements between different molting wetlands and the transmission rate were the key predictors of HPAI perpetuation. While wetland extent was not a significant predictor of HPAI perpetuation, it had a large effect on the number of infections and associated death toll. Our results indicate that conserving undisturbed habitats for wild waterfowl during the molting stage of the breeding season could reduce the risk of HPAI transmission.

## 1. Introduction

Highly pathogenic avian influenza (HPAI) is endemic in Southeast Asia and presents a constant risk to the health of both humans and wild birds. For instance, from January 2003 to December 2024, there were 261 laboratory-confirmed cases and 142 deaths [1] from H5N1 infections in humans in the Western Pacific (specifically China, Cambodia, Lao PDR, and Vietnam). Meanwhile, the emergence of clade 2.3.4.4b has had far-reaching global implications for hundreds of avian species [2]. Thus, it is critically important to understand the mechanisms by which HPAI persists and spreads so that areas of greatest risk can be identified, and prevention and response resources can be best applied [3,4]. Extensive research over the past two decades has dramatically improved our understanding of HPAI transmission dynamics in Southeast Asia, highlighting the potential for wild waterfowl to mediate the long-distance spread of HPAI both at regional and intercontinental scales [5,6,7] and identifying periods of spatiotemporal overlap between wild birds and domestic poultry [8,9,10]. However, while the examination of HPAI in market chains has identified mechanisms allowing the persistence of HPAI in domestic poultry [11,12,13], there are few data available on the factors driving or enabling perpetuation or persistence across the unique life stages that define the annual cycle of waterfowl.

Seasonal dynamics of host–pathogen interactions, habitat use, and environmental conditions are important ecological factors that influence the risk of infectious disease transmission differently across the annual cycle [14]. However, studies of HPAI transmission by waterfowl, associated risk assessments, and transmission-relevant host behaviors have not been distributed equally across stages of their annual cycle. The existing literature has mainly focused on wintering grounds where the density of both poultry and human populations is high [10,15]. Similarly, the spring and fall migration stages have been studied due to concerns of migrant-mediated transmission across habitats [16,17,18], although the role of populations intermixing at staging and stopover areas in disease dynamics remains poorly understood [19]. While serological sampling has been conducted to document the prevalence of HPAI and the immune responses of waterfowl during the breeding season [20,21], the dynamics of amplification, perpetuation, and the spread of viruses introduced to breeding grounds have rarely been investigated [22].

The lack of data regarding the ecological factors driving HPAI transmission dynamics on the breeding grounds is a notable weakness in the current literature, especially given the unique behaviors waterfowl exhibit during this period and the potential they present for HPAI spread. Waterfowl are typically found in high densities at wintering sites, but they typically distribute widely and at low densities while breeding. However, during the stage known as the molt migration, which occurs in the late breeding season, waterfowl from disparate breeding sites congregate at a limited number of wetlands that provide adequate food and protection from predators while birds molt their flight feathers [23,24]. The molt migration behavior results in a mixture of populations from different geographic origins forming loosely connected meta-populations until their flight capability is regained post-molt. This stage presents a major opportunity for HPAI viruses to spread among formerly distant individuals since they are constrained to the same habitat and especially because naïve juveniles are present, for whom this aggregation may represent the first exposure to these viruses [25,26]. Furthermore, the molt of remigial feathers has substantial energetic and nutritional costs [27], potentially leading to molt-mediated immunosuppression. Such immunosuppression could lead to HPAI outbreaks similar to those reported for migrating birds [28], although the relationship between lower immunity and HPAI outbreaks is not well established [22].

Despite the high potential for HPAI transmission during the molt migration stage, the existing literature has not supported the occurrence of large outbreaks nor the persistence of HPAI in this period. For instance, HPAI infection from molting to pre-migration was not supported in juvenile Pink-footed Geese (*Anser brachyrhynchus*) [14], and the lack of HPAI outbreaks in the late breeding season in Mongolia suggests that waterfowl are not continuous reservoirs of HPAI infection [20]. One possible explanation for the lack of regular HPAI perpetuation through the molt migration stage is that the flightless period is of sufficient duration that outbreaks run their course within isolated molting sites before extensive intermixing can occur once waterfowl regain their flight feathers [29]. However, we have very little understanding of individual movement behaviors during the molt migration that may allow for the perpetuation of HPAI transmission and whether climate change effects on their environment may alter that possibility.

In this study, we hypothesized that HPAI viruses introduced into the breeding grounds of waterfowl must persist through the molting stage for the perpetuation of the fall migration. To test this hypothesis, we used a simulation model to examine how waterfowl behavior and ecological conditions during this period affected transmission and persistence. We used an integrated agent-based model (ABM) [30] that included susceptible–exposed–infectious–recovered (SEIR) states and applied empirical field data from Swan Geese (*Anser cygnoides*) in northeastern Mongolia to create ecological and epidemiological simulations. We aimed to (1) apply telemetry data to describe the spatiotemporal movements of Swan Geese in the late breeding season, (2) estimate the chance of HPAI perpetuation until the fall migration, and (3) assess the role of host, environmental, and disease parameters in HPAI infections.

## 2. Materials and Methods

### 2.1. Study Area

**Figure 1:**
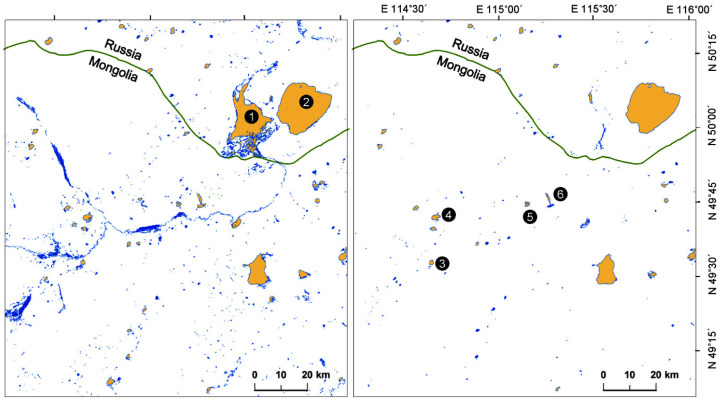
Study areas in Mongolia and Russia showing the potential molting wetlands (≥1 km^2^; orange) and other rivers and smaller wetlands (blue) in the region. The left panel shows the wetland mosaic in a wet year (July 2014) and the right panel shows the wetland extent in a dry year (July 2015). In these panels, lakes are represented with yellow polygons, and notable locations including two major lakes (1: Barun-Torey Lake and 2: Zun-Torey Lake) and four goose-capture sites are indicated (3: Chukh Lake, 4: Khaichiin Tsagaan Lake, 5: Bus Lake, and 6: Galuut Lake) with numbered black circles.

Our study area is the transboundary region in northeastern Mongolia, where Dornad Province borders Zabaykalsky Krai in Russia’s Transbaikalia region (Figure 1). As part of the Mongolian–Manchurian Steppe, also known as the Mongolian–Manchurian Grassland, this area is one of the most intact grasslands remaining on Earth [31]. Flooding conditions are highly variable in the study site, occasionally causing the loss of >90% of lakes and small rivers in dry phases [32]. Despite their unpredictable extent, the wetlands of the Mongolian–Manchurian Steppe are important breeding habitats for Swan Geese [31,33] as well as other native waterfowl species [34,35,36]. However, the Mongolian–Manchurian Steppe is under threat from a litany of stressors including overgrazing, mining, and rapid climate change [37].

### 2.2. Study Species

The Swan Goose is an endangered migratory waterbird endemic to East Asia [31], which connects the wintering grounds in south China and breeding grounds in Mongolia and Russia [31,33,38]. The estimated global population of the Swan Goose is 36,000–43,500 but is decreasing [31]. Major wintering grounds of Swan Geese are Poyang Lake and the Yangtze River watershed in South China [39,40], the epicenter of HPAI emergence and outbreaks due to the high density of poultry, humans, and wintering wild waterbirds [41,42]. Continued habitat loss and concentrating populations at fewer wintering wetlands have likely increased the susceptibility of the Swan Goose to HPAI [31], which increases the potential for this species to serve as a vector along the East Asian Flyway similar to the suspected role of the Bar-headed Goose (*Anser indicus*) in the Central Asian Flyway [43]. Although the behaviors and breeding biology of the Swan Goose are not fully described, recent telemetry and marking studies provided key ecological information on their habitat use and migration [44,45,46,47] to parameterize details for our modeling effort.

### 2.3. Documenting Wetland Extent

It is unknown how Swan Geese select molting areas, and little information on the distribution of their molting wetlands is currently available. However, large water bodies provide important habitats where molting geese may avoid predators [23]; therefore, understanding the abundance and distribution of large wetlands in the late summer was included in our modeling efforts. We collected satellite images (WRS path: 126, WRS Row: 25–26) provided by the Landsat 8 Operational Land Imager (OLI) sensor on 23 July 2014 and 10 July 2015 and used the modified normalized difference water index (MNDWI) [48] to construct two layers (1303 × 1426 pixels, resolution: 100 m × 100 m) indicating the size and distribution of available wetlands (Figure 1). We used the 1.0 km^2^ threshold to identify potential molting lakes that may provide abundant food resources and a safe habitat for flightless geese.

The 2014 breeding season had above-average rainfall, and 763.6 km^2^ of large wetlands including 41 lakes (596.2 km^2^, 78% of total wetlands) were identified in the study area in July. This decreased to 393.4 km^2^ with only 24 lakes (93% of total wetlands) in July 2015, which was a particularly dry year in this region (Figure 1). Barun-Torey Lake went from 167 km^2^ in 2014 to <6 km^2^ in 2015 (Figure 1), causing a massive loss of habitat for waterbirds similar to conditions reported in July 2009 [49]. The four sampling sites where we captured molting geese (see capture and telemetry) were identified as lakes with molting birds in both years.

### 2.4. Capture and Marking

We worked with local experts to survey major wetlands in our study area to locate large flocks of pre-molt Swan Geese during the late breeding season. Capture and marking information are summarized here but also presented elsewhere [45,46]. Once flocks were identified, we set up corral traps and lead nets along lake shorelines and deployed kayaks and motorboats to capture or herd flightless geese into the corral traps [33]. From 27 July to 31 July 2014, we captured birds at Bus Lake (49°44′ N, 115°09′ E), Galuut Lake (49°44′ N, 115°17′ E), Chukh Lake (49°31′ N, 114°39′ E), and Khaichiin Tsagaan Lake (49°41′ N, 114°40′ E) (Figure 1). Upon capture, we measured, sexed, and aged juveniles and molting adults, and we then marked each bird with a unique numbered collar band and metal leg band. We also took paired blood samples and cloacal swabs from 59 geese for HPAI virus detection and stored the samples in liquid nitrogen until laboratory analyses were performed at the Southeast Poultry Research Laboratory, U.S. National Poultry Research Center, U.S. Department of Agriculture (USDA), Athens, Georgia. An analysis with RT-PCR confirmed that H5 clade 2.3.4.4 was detected in 3 out of 57 tested cloacal samples (2 samples were lost to contamination), indicating a 5.26% (1.1–14.6%, 95% C.I.) prevalence of HPAI in the molting geese. Additionally, we deployed 49 transmitters that used the global positioning system (GPS) and Global System for Mobile Communications (GSM 20–70, Microwave Telemetry, Inc., Columbia, MD, USA) in duty cycles varying from 1 to 360 min intervals. All transmitters were placed on the birds’ backs and secured via Teflon harnesses or neck collars [50], with the harness–transmitter package being <2.5% of the body mass of the goose (<60 g). We examined the location data from 49 marked birds after deleting 12 individuals with an insufficient number of locations collected prior to departure from the study area to effectively determine their movements, leaving 37 birds for analyses.

### 2.5. Determining Movement Phases

An earlier study and preliminary review of our telemetry data indicated that the median date of Swan Goose arrival to molting areas was 21 June [33] with the final departure from the study area in September as birds either initiated southward migration or moved to pre-migration staging areas. We used the median date for the first period of our study window, since the birds were still flighted as they arrived, and some could be moving through to other sites for molting. Thus, we set our study window to be from 21 June to 31 August and divided this window into three distinct periods based on flight ability and habitat use: molting, post-molting, and pre-migration. We considered geese as having entered the molting stage upon arrival to their respective molting area. We determined the date that each goose first recorded an inter-wetland movement, defined as travel greater than 5.5 km (the longest axes length of four molting lakes where the geese were marked) from its initial marking site. We used our general departure date (31 August) as the date of the first inter-wetland movement for any individual that lacked inter-wetland movements. We then used the earliest and mean date of their first inter-wetland movement to determine the transition from molting to post-molting and from post-molting to pre-migration periods.

### 2.6. Family Groups as Agents

Geese generally stay in family groups and goslings stay with adults throughout the breeding and molting season; thus, we considered a family as a single experimental unit (hereafter agent) that shares the same infection state. Unfortunately, there is a lack of demographic information for this species in this region such as the total population, number of breeding pairs, age ratio, and family structure. For instance, the estimated population of 3500 could be represented by anything from 3500 agents, assuming entirely non-breeding individuals, to 583 agents if all birds were part of an average family unit composed of two parents and four goslings [51]. Therefore, we ran simulations with either 1000 or 2000 agents, which allowed us to examine the impact of increased host density while balancing the uncertainty in the demographics of this population.

### 2.7. Rules of Movement Behavior for Agents

We used four basic movement rules for individuals within our ABM framework on the basis of our empirical telemetry data and general waterfowl movement ecology literature.

Rule 1: The local movements of agents follow the patterns identified by telemetry data. We used our telemetry data to model the local movements of each agent. We selected locations from geese that recorded fixes at least hourly to identify the local movement patterns (*n* = 13 geese) and calculated the mean hourly displacement (i.e., the linear distance between locational fix at hour 1 to hour 2, hour 2 to hour 3, etc., averaged per bird) in each of the three periods (molting, post-molting, and pre-migration) to use as the typical local movement pattern. We found that the distance of hourly displacements and inter-wetland movements followed an exponential decay (see Results). We used random numbers generated from an exponential distribution of mean distances to simulate the hourly and inter-wetland movements of each agent.

Rule 2: The agents stay within the wetlands. A review of our telemetry data indicated that Swan Geese almost exclusively utilized wetland habitats and not the uplands throughout the study period. Thus, we required that whenever the agents were outside of the wetlands at the beginning of the simulation or at any point during the simulation, they returned to the wetlands. In instances where agents were not already at molting lakes at the start of the simulation, they moved toward the nearest molting lake at a relatively slow speed (≤1 km/h) that was determined from the tracking data. This movement rate was applied for the first five days of simulations to represent the asynchronous and delayed arrival of geese to molting lakes. Following the initial travel period during which 99% of agents arrived at molting lakes, agents located outside of wetlands returned to the nearest wetland at a greater speed that varied with the distance from the wetlands (i.e., 10.6 km/h at 10.6 km away from the nearest wetlands, but ≤1 km/h at <2 km distant). To allow for the depiction of wetlands within our simulation, we developed maps based on wetland extent layers representing distance gradients to molting lakes and all available wetlands by period. Agents moved along the distance gradients with a flexibility of 15° in direction from their movement path. We then compared ABMs between 2014 (a wet year) and 2015 (a dry year) to depict the differences in agent movements with a loss of wetlands from likely climate change effects such as prolonged drought.

Rule 3: Agents perform occasional inter-wetland movements. Agents sometimes demonstrated distant movements between different wetland patches, especially in the post-molting and pre-migration stages. Although infrequent, inter-wetland movements were documented and enabled intermixing that may play a role in HPAI virus transmission between isolated molting populations. We reviewed the movements of 37 tracked geese and identified their inter-wetland movements; however, we did not include movements within the same or linked wetland patches or round trips where geese returned to the same patch within 48 h regardless of their distance moved. We excluded these movements to ensure erroneous telemetry fixes were not included; thus, our results represented a conservative measure of potential transmission among wetlands. We then estimated the daily rate of inter-wetland movement (%) by dividing the number of qualifying events by cumulative telemetry days (number of birds x tracked days) in each stage. To model these inter-wetland movements, we displaced the agent in a random direction over a random distance (bounded by the range of inter-wetland movement distances determined from the telemetry data for the relevant period), and we let the agent return to the nearest wetland from the relocation site following Rule 2. While rates of movements within the post-molting and pre-migration stages were based on the telemetry data, we allowed three different movement scenarios for agents in the molting stage. The first scenario reflected our telemetry data (see Results) that included no inter-wetland movements (0%) because the geese were flightless during this period. However, to explore the potential for transmission in years when movements were asynchronous, we allowed for simulations with very weak inter-wetland movements (1% or 2%).

Rule 4: Agents demonstrate flocking behavior. Since Swan Geese are gregarious [31], we used a flocking model [52] to simulate the interaction between agents. Each agent moves forward at its own speed but follows three behaviors affecting the agent’s heading: alignment, cohesion, and separation [52]. The visual range was set to a 1-km radius to find and interact with the nearest agent for alignment and cohesion. An agent tended to turn in the same direction as its flock mates and moved toward other nearby agents. We also set a 5-m distance for separation, which meant that an agent turned to avoid another bird to maintain the minimum separation. If there were no nearby agents, each agent followed a random heading for the next hourly movement. We assumed that agents changed their heading no more than 30°, and the change in their heading was random within the limit set for flocks.

### 2.8. Comparability of Simulated Movements

To enable a comparison between the movements generated via simulation rules with those of the tracked birds, we used NetLogo (version 5.3) software [53] to perform simulations with one-hour intervals from 21 June to 31 August. These exploratory simulations followed the rules defined above, but tracking data were not available during a dry year. Additionally, simulations for home range validation were performed assuming no mortality and with daily rates of inter-wetland movement during the molting stage set to zero. We performed 80 simulations split across four scenarios (20 simulations per scenario). In these scenarios, birds could be distributed at the beginning of the simulation (1) randomly within the region or (2) at one of the four marking lakes. All simulations had either 1000 or 2000 initial agents, which would be an expected number of individuals for a region of this size [51].

Following the completion of simulations, we took hourly locations for four randomly selected agents from each simulation and used a location-based kernel density estimator to estimate seasonal home ranges for each of our 37 tracked birds. As a non-parametric and probabilistic method that calculates home range boundaries based on the complete utilization distribution (UD; [54]), kernel-based home range estimation is highly sensitive to the bandwidth value (h) used [55,56]. Because of the large variance in sample sizes, time intervals, and spatial extents of location fixes across our 37 tracked geese, the reference bandwidth (h_ref_) and the least squares cross-validation bandwidth estimation (h_lscv_) generated over-smoothed home ranges with larger bandwidth values for our GPS dataset [56]. To minimize the variance across individuals, we applied the plug-in bandwidth (h_plug-in_) selection method with bivariate bandwidth vectors (h_x_ and h_y_) in the R package ‘ks’ [57]. The two derived bandwidths for each of the 37 geese were averaged into a single numeric value to produce a standardized smoothing bandwidth (h = 520 m), and we applied this standardized value to the kernel home range estimation for both tracked geese and simulated agents. Because inner density isopleths tended to provide less biased estimates of home range area, we selected the 90% isopleth for home range estimates [54]. We compared 320 simulated home range sizes with the 37 home ranges from our empirical data with a non-parametric Kruskal–Wallis test. We also calculated a static interaction index for each of the 666 pair combinations or dyads of the tracked geese, and that value was used as the proportion of home range overlap between two birds of a dyad [58]. The static interaction index was used as an indicator of the potential for dynamic interaction in dyads [59].

### 2.9. SEIR States and Disease Transmission

After we confirmed that our simulation rules were sufficient to produce movement patterns comparable to those observed in tracked geese, we introduced disease parameters into our ABM via a susceptible–exposed–infectious–recovered (SEIR) model, the common compartmental model describing the transmission of an infectious disease [60]. Unlike the SEIR model describing epidemics at a population level, ABM tracks infection history in epidemiological studies by exploring the heterogeneity of each agent and adds up all individual disease states at each step of the simulation to describe overall disease transmission dynamics [61]. We adopted four SEIR states to represent an individual’s disease state at a given time (Figure S1), and because of the short simulation period, we used a closed population for the ABM without demographic changes from birth, death, immigration, or emigration, except for the mortality of infected birds.

When susceptible agents contacted infectious ones, they turned to exposed agents according to the chance of transmission per contact. To allow for the simulation of contact (including both direct and indirect), we calculated 88 minimum convex polygon (MCP) home ranges for an hour period based on 5069 location fixes collected every minute from 11 Swan Geese. The mean MCP area was 8138 m^2^, ranging from 26 m^2^ to 79,840 m^2^, which was equivalent to a circular home range with a radius of 51 m (Table 1). Thus, any birds occurring within 51 m of an infectious bird were considered exposed and at risk of infection. Following exposure, we assumed that 4.4 ± 0.4% (low) to 9.7 ± 1.0% (high) of the total contacts resulted in infection (Table 1). These values were based on previously established influenza A virus transmission rates [62,63], because no data about the contact probability of HPAI transmission are available for free-ranging wild geese. To determine the initial number of infectious geese, we iteratively assigned integers of 50, 100, and 200 agents to represent 2.5–20% of the number of total agents as infectious and assigned the remaining agents as susceptible.

If an agent does contract avian influenza, it transitions to the infectious state and becomes infectious after a latency period. Since no studies have been conducted to assess the pathogenicity of H5N1 in Swan Geese, we set a mean latency period of 2.5 days with a standard deviation of 0.5 days (Table 1) based on captive trials with Cackling Geese (*Branta hutchinsii*) and Bar-headed Geese [64]. Similarly, we set the infectious period as 5.7 ± 1.4 days based on the minimum and maximum duration of virus excretion in three conspecific goose species [65]. Within our model, we used gamma-distributed latent and infectious periods, as they are believed to be more realistic for most infections than the classical exponentially distributed model [66]. Our model did not include the effects of infection on the movements of agents, so the infected agents moved the same way as uninfected agents. While active infection may have effects on movements, these effects appear to be species-specific [67,68,69], and no such data are available for Swan Geese.

Following infection, agents must either transition to the recovered state or suffer mortality. The mortality of HPAI-infected birds may vary by factors such as species [70,71], age [72,73], and previous exposure to different viral strains [74,75]. Although infected birds died in many laboratory experiments [65], two of three Swan Geese that tested positive for H5N1 in our sampling efforts recovered and successfully completed migration. Thus, we used a mortality rate of 33.3% for this study (Table 1) as it reflects the limited field data available for this species. We assumed that the HPAI strain introduced to molting lakes was a novel virus to those geese, and so all agents were considered naïve, meaning that all exposed individuals were equally likely to transition from exposed to infected and from infected to either recovered or died. Additionally, recovered individuals became immune to the viral strain for the duration of this short-lived simulation. It should be noted that homo- and heterosubtypic immunity, of varying degrees, to H5N1 has been demonstrated in waterfowl and could lower the likelihood of viral persistence depending on other system conditions [75]. By assuming host naivety in this study, we were maximizing the potential for cross-seasonal persistence, but these factors could be explored in future work.

#### Final Simulation

We simulated the hourly movements of agents from 21 June to 31 August (1728 h) and performed 200 iterations for each of the 72 parameter combinations (2 host densities × 3 daily movement rates × 2 water years × 3 initial prevalence rates × 2 transmission rates), resulting in a total of 14,400 simulation runs (example parameterization provided in Supplementary Text S1). Agents were randomly distributed across the arena at the start of all 14,400 simulations. We assumed that HPAI perpetuated fall migration when one or more agents were either in an exposed or infectious state at the end of the simulation. We determined the number of models that resulted in HPAI prevalence values comparable to the proportion observed in our field sampling (3 of 57 or 5.26%) on day 36 (27 July, when we began our HPAI field sampling) for 7200 runs from 36 scenarios applying the 2014 wetland mosaic. For each model, we calculated the mean HPAI prevalence and 95% confidence interval by taking the number of infectious agents divided by the number of live agents at the sampling date. The ABM outputs from our 14,400 simulation runs were analyzed with a generalized linear model (GLM) to document the effect of parameters on HPAI perpetuation and infection profiles. We used a logit link to analyze the binomial perpetuation rate, Poisson distribution with a log link for non-negative integers such as the number of infected agents, and gamma distribution with a log link for continuous and positive values such as length of persistence in a GLM test. We used R software 3.1.1 [76] for all statistical analyses.

## 3. Results

### 3.1. Movements During the Molting Stage

**Figure 2:**
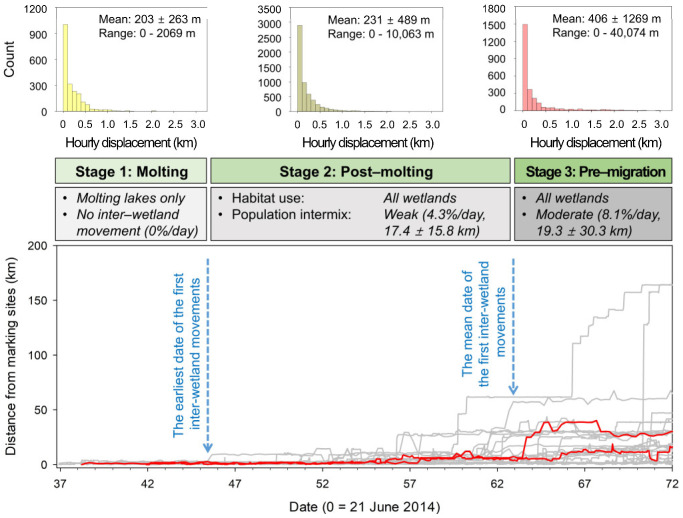
Changes in the distance of Swan Geese (*Anser cygnoides*) from their own capture and marking sites over time during the late breeding season in 2014. Bold red lines represent HPAI-infected geese, while gray denotes the other geese. Three stages were separated based on the earliest and the mean dates of the first inter-wetland movement detected in each tracked Swan Goose. The pattern of habitat use and inter-wetland movement is shown above the stages.

A total of 28 out of 37 geese used wetlands outside of their molting lake over the course of our study. The earliest date of the first inter-wetland movements was 45.7 days (the afternoon of 5 August) and the mean date was 62.9 days (the night of 22 August), which we used to separate the molting stage into three periods (Figure 2). While no inter-wetland movements were observed during the molting period, we documented that 4.3% of movements or 17.4 ± 15.8 km inter-wetland movements occurred during the post-molting period and that 8.1% of movements or 19.3 ± 30.3 km occurred in the pre-migration period (Table 2). The hourly displacement in the three periods calculated from 13 tracked geese with hourly fixes were 203 ± 263 m (*n* = 2066), 231 ± 489 m (*n* = 5694), and 406 ± 1269 m (*n* = 2735), respectively (Table 2). Although their hourly displacements showed a slight increase over time (linear coefficient = 7.7 m/day, r = 0.094, *p* < 0.001), it was not significantly different between the three periods (Kruskal–Wallis test; H = 2.826, df = 2, *p* = 0.243).

### 3.2. Home Range Comparisons

**Figure 3:**
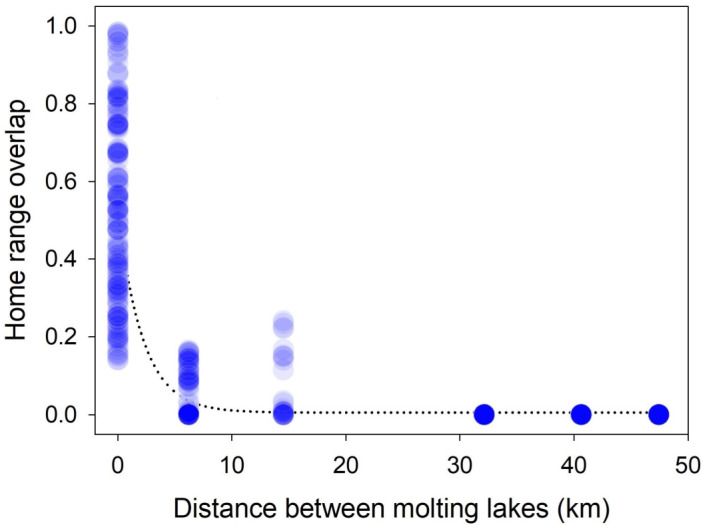
Relationship of the home range overlap measured as static interaction index between 666 dyads from 37 Swan Geese (*Anser cygnoides*) as a function of the distance between their molting lakes in the late breeding season. Dashed line represents an exponential fitting curve from nonlinear regression.

The estimated home range size from tracked Swan Geese was 22.7 ± 15.4 km^2^ (mean ± SD; range: 6.6–65.3 km^2^) and not significantly different from the home range size estimated for agents in the models (28.9 ± 30.9 km^2^; range: 7.5–208.1 km^2^, H = 3.064, df = 4, *p* = 0.547; Figure S2). The mean overlap of home ranges for 666 dyads was 0.154 (range: 0–0.984). The static interaction between two geese molting at the same lake (mean: 0.521, range: 0.141–0.984, *n* = 184 dyads) was significantly greater than the dyads that molted at different lakes (mean: 0.014, range: 0.000–0.241, *n* = 482 dyads) (Mann–Whitney U Statistic = 37,757.0, *p* < 0.001). In particular, the exponential decline in indices by distance (r = 0.869, *p* < 0.001) indicated that home ranges generally overlapped between birds in the same lake, whereas the proportion of overlapping home ranges rapidly decreased as the distance between molting lakes increased (Figure 3).

### 3.3. Perpetuation of HPAI to the Fall Migration

Across our 14,400 simulations from 72 models, the mean length of HPAI persistence was 55.5 ± 8.4 days (range: 30.7–72.0 days) with perpetuation to the fall migration of one or more HPAI infectious agents occurring in only 360 simulations (0.25%). Thus, we found a low probability of HPAI perpetuation from the molting stage to the fall migration (Figures S1 and S2). When perpetuation occurred, 0.1% of the surviving agents were in exposed or infectious states (1.6 ± 1.2 out of 1559 ± 98 surviving agents, range: 1–7). The HPAI prevalence at day 36 (5.26%) fell into the 95% confidence intervals of only 2 of 36 models that used the wetland mosaic for wet years (2014) (Table 3), and both of these models included 2000 agents and 50 initial infectious agents. These two models had inter-wetland movement rates of 1% and 2%, despite the lack of inter-wetland movements in our tracking datasets. These two models indicated that HPAI persisted for 65.7 ± 4.8 and 63.3 ± 5.7 days with a 13% or 8% chance of perpetuation to the fall migration (Table 3).

### 3.4. Effects of Parameters on HPAI Infection Profiles

**Figure 4:**
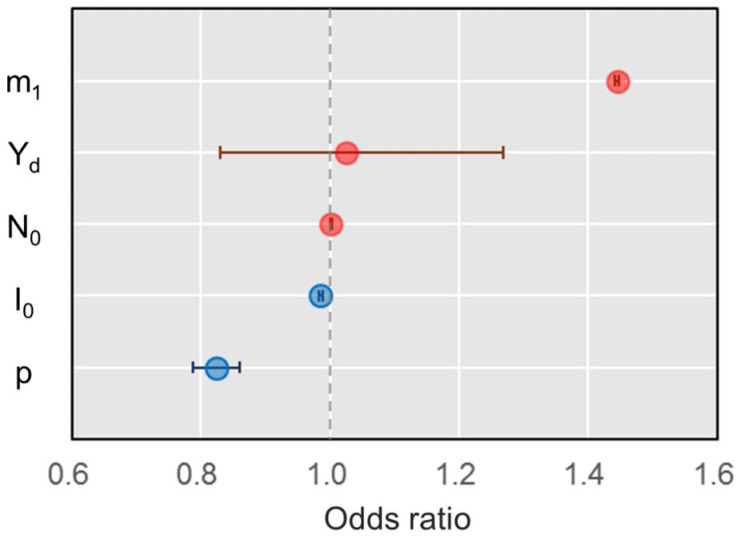
Odds ratio of five factors affecting the chance of HPAI perpetuation until the following migration season in Swan Geese (m1: daily rate of inter-wetland movement in the molting stage, Yd: dryness, N0: Initial number of agents, I0: Initial number of infectious agents, p: transmission rate). Red and blue dots denote positive and negative means, and horizontal bars indicate 95% confidence intervals (95% CI).

**Figure 5:**
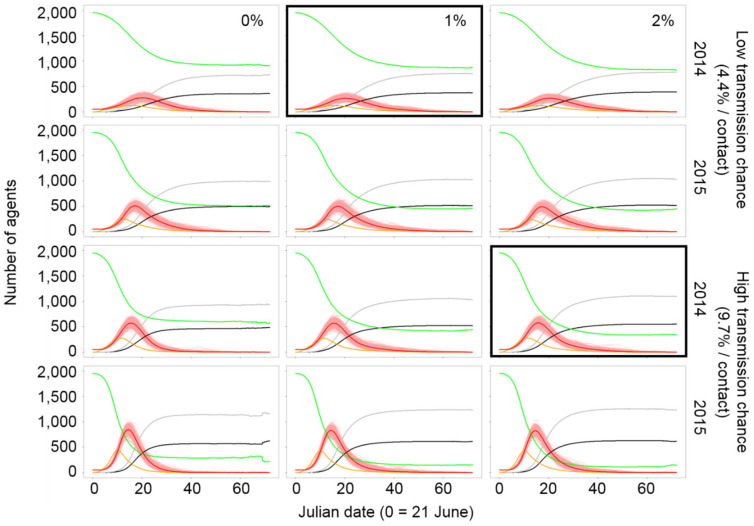
Infection profiles of the two models (marked bold) reflect our observed prevalence rate along with similar models sharing initial conditions (2000 agents with 50 infections). Profiles were simulated from 200 iterations in the late breeding seasons of 2014 (wet year) and 2015 (dry year). Red lines indicate the mean number of infectious agents calculated from 200 individual infection profiles marked by fine, overlapped pink lines. Green, orange, gray, and black lines denote the mean numbers of susceptible, exposed, recovered, and dead agents, respectively. Percentage in top right of each column means the daily probability of inter-wetland movement of Swan Geese (*Anser cygnoides*) in the molting stage (left column: 0%, center: 1%, right: 2%).

Our GLM test results indicated that four out of five parameters were significant predictors of HPAI perpetuation (Figure 4). The daily rate of inter-wetland movement in the molting stage (χ^2^ = 29.76, *p* < 0.001) and the transmission rate (χ^2^ = 81.71, *p* < 0.001) were related to perpetuation. The initial number of total agents and infectious agents were also weak but significant predictors of perpetuation. On the other hand, dry conditions were not a predictor of perpetuation because of the large variation (χ^2^ = 0.05, *p* = 0.831). Greater rates of inter-wetland movements in the molting stage increased the chance of HPAI perpetuation by causing longer persistence with more infections and dead agents, but it lowered the peak number of infectious agents without altering the timing of outbreaks (Figure 5, Figures S3 and S4). Dry conditions resulted in earlier outbreaks and shortened the length of HPAI persistence. Although it did not affect the chance of HPAI perpetuation in the late breeding season, Dry conditions strongly increased the overall and peak number of infections, resulting in higher total mortality. A higher transmission rate caused earlier outbreaks and resulted in a greater number of overall and peak infections. More agents died but the risk of perpetuation decreased with the shorter period of persistence. A larger number of agents increased the chance of perpetuation, peak and total infections, and deaths. More infectious agents introduced at the beginning of the period reduced perpetuation since outbreaks occurred earlier and resulted in more infectious and dead agents.

## 4. Discussion

Our models simulated the duration of HPAI persistence in a population of Swan Geese following the introduction of the disease in the molting stage and examined the factors that influenced perpetuation to the fall migration. Overall, our models indicated a low probability of HPAI perpetuation to the fall migration, but the two models that most closely matched the HPAI prevalence rate that we observed in the field showed that perpetuation might occur. Additionally, the agents in exposed and infectious states at the end of our simulations were only 0.1% of the surviving agents, and this is far below the 2.5% of agents (50 infections, 2000 agents) that were used as initial conditions for the two models. However, a low prevalence rate may still result in transmission during the fall migration, because Swan Geese form large migratory flocks composed of an admixture of populations that include adults and juveniles naïve to HPAI. Also, geese may have a weaker immune response during the subsequent fall migration due to the demands of migration [28,77].

The likelihood of HPAI perpetuation is influenced by several factors, but our modeling results indicated that movement between wetlands during the molting stage was a key predictor of HPAI perpetuation. Our telemetry data showed that molting Swan Geese moved very little within their primary molting lakes during the molting stage, likely because they were flightless for several days following their molt [31]. The greatest overlap of home ranges occurred between individuals molting in the same lake, with an exponential decrease in overlap observed as the distance between molting lakes increased. This implies that a greater risk of transmission occurs between birds in the same molting lake [78] with limited opportunity for infection to spread between populations at different lakes [20]. However, the models that best fit the observed HPAI prevalence rate had inter-wetland movements during the molting period.

One explanation for the apparent contradiction in the relationship between inter-wetland movements and prevalence rate in the empirical and modeling results may be that larger movements occurred prior to when we captured and marked the Swan Geese. Also, inter-wetland movements may be relatively rare, and although we marked a relatively large sample of birds for a telemetry study, we may have had insufficient numbers to capture the infrequent movements which may be expected if they are driven by unpredictable disturbance events (i.e., human activity and wildfire). Movements may be driven by asynchronous molt (i.e., an increased number of non-breeders) or unsuitable conditions early in the season (i.e., predators and a lack of resources). Alternatively, while our models determined HPAI persistence and perpetuation from a single species, transmission between wetlands could have been driven by other waterbird species. While the Swan Goose was the sole breeding waterbird species in some lakes, other breeding birds including dabbling ducks (*Anas* spp.), shelducks (*Tardorna* spp.), and other waterbirds (such as gulls, herons, cormorants, and shorebirds) also occurred in the study area [79]. A recent study suggests that HPAI epidemics are strongly correlated with the waterbird community attributes [80]. Thus, models reflecting inter-wetland movements may have been capturing the complexity of a multi-species environment that was not explicitly addressed.

The second primary factor driving perpetuation in our models was the transmission rate. Based on model comparisons, we demonstrated that more infectious HPAI strains may cause more infections, more dead birds, and rapid but shorter outbreaks. While the absolute values that we used here are not drawn from studies of H5N1 in wild birds, the differences observed when we varied the transmission rate indicate that this component is very important. New strains of HPAI that only cause small epidemiological and demographic differences may greatly affect the perpetuation of the disease.

Habitat loss was expected to exacerbate pathogen spread [81], but contrary to our expectations, dry or wet conditions were not a statistically significant predictor of HPAI perpetuation. HPAI perpetuation did span a wider range (2.8–18.0% versus 9.2–14.0%) when models that matched our empirical prevalence values were applied to the wetland mosaic in a dry year, implying that drought may increase the uncertainty of lower or higher HPAI perpetuation. However, the wetland mosaic had a major effect on outbreak size and mortality. During dry conditions, birds were confined into fewer wetlands, which increased relative host densities and contact probability and resulted in earlier outbreaks with more infections and a higher associated death toll. This result suggests that reductions in available wetlands will magnify the effects of HPAI outbreaks on wild waterbird populations. This is especially concerning given the multitude of threats currently facing wetlands in this region. For instance, the recent drought during the past decade resulted in the hottest temperatures in the past 1000 years in central Mongolia, and episodic future droughts are expected to occur in Inner Asia regions [82,83,84,85,86]. Also, rapid socio-economic changes have resulted in numerous human-caused habitat disturbances such as overgrazing, mining, irrigation, and embankments [32,85,87]. In light of these future threats, HPAI represents a notable demographic vulnerability for Swan Geese, which are already experiencing declining populations [31,47,88] and a low survival rate [44].

Our findings suggest that conservation efforts in molting regions of Swan Geese could ensure that there is a healthy mosaic of undisturbed molting wetlands. If numerous sites are available, the overall population within a region will be split into a greater number of subpopulations, diluting the number of birds at risk of mortality with each disease introduction. Also, management activities aimed at minimizing human disturbance and reducing predators during this vulnerable stage could reduce the number of non-breeding birds that disperse to other molting lakes earlier than family groups [51], thereby reducing the potential for viral transmission between wetland sites.

Our study used an integrated ABM approach that included a SEIR-based transmission model and empirical data from wild Swan Geese to highlight how the unique ecology of a wild population during a specific life stage may influence HPAI dynamics. Integrated ABM modeling approaches have been used successfully to develop scenarios for other life stages including migration [81] and for other diseases including the West Nile Virus [89]. For our specific case, we found that the perpetuation of HPAI in Swan Geese may be related to specific movements and habitat conditions during the molting stage. In addition, our data suggest that the potential for more transmissible HPAI strains, coupled with threats to wetland habitats, will likely increase challenges to sustaining migratory waterbirds in this region. However, protecting molting habitats and reducing disturbance may lower disease risks and sustain waterbird populations in areas with endemic HPAI infections.

## Figures and Tables

**Table 1:** Variables used in the agent-based model for HPAI virus persistence among Swan Geese (*Anser cygnoides*) in the late breeding season.

Parameters	Value (Unit)	Descriptions and Sources
**Host**		
Initial number of agents	1000 and 2000 agents	Number of agents representing 3500 Swan Geese based on family group sizes [51]. Baseline integers assigned in an effort to balance the uncertainty in the demographics of this population.
Daily rate of inter-wetland movement	Molting: 0%, 1%, 2%	The rate during the molting period increased from 0% (telemetry data) to 2% to explore model sensitivity, while post-molting and pre-migration were based on telemetry data.
	Post-molting: 4.3%	
	Pre-migration: 8.1%	
**Environment**		
Arena	Grid matrix: rows = 1430, columns = 1303, resolution: 100 m × 100 m	Pre-defined study area (143.0 km × 130.3 km), which was identified from the boundary of telemetry data.
Distance from wetlands	Dependent on location in arena	Two rasters of the Euclidean distance to the closest molting lakes (molting) and available wetlands (post-molting and pre-migration) for each year.
**Pathogen**		
Initial number of infectious agents	50, 100, or 200 agents	Iteratively assigned integers to represent 2.5–20% of the total number of agents for the ABM.
Latent period	2.5 ± 0.5 days	Fixed value (mean ± SD) estimated from experimental studies using captive geese [64]. Transformed into gamma-distributed latent period [alpha = mean^2^/variance; lambda = 1/(variance/mean)] for ABM in NetLogo [53].
Infectious period	5.7 ± 1.4 days	Fixed value (mean ± SD) estimated from experimental studies with captive geese [65] transformed into gamma-distributed infectious period [alpha = mean^2^/SD^2^; lambda = 1/(SD^2^/mean)] for ABM in NetLogo [53].
Contact distance	51 m	Estimated from the mean radius of hourly MCP areas of 37 tracked geese in this study.
Transmission rate	4.4 ± 0.4%, 9.7 ± 1.0%	Baseline integers and their 10% of standard deviations assigned from the estimated transmission rates of avian influenza virus of close contacts in human households: 4.4% [62] and 9.7% [63].
Mortality rate	33.3%	Apparent mortality documented from telemetry data in this study. Two out of three HPAI-infected geese survived and migrated.

**Table 2:** Movements of Swan Geese (*Anser cygnoides*) determined from the telemetry study over three stages of the late breeding season.

	Molting (21 June–5 August)	Post-Molting (6 August–22 August)	Pre-Migration (22 August–31 August)
Inter-wetland movement			
No. events (A)	0	27	27
No. tracked birds (B)	37	37	37
No. days (C)	46	17	9
No. telemetry days (D = B × C)	1702	629	333
Daily rate (E = A/D × 100)	0.0%	4.3%	8.1%
Distance (Mean ± SD)	--	17.4 ± 15.8 km	19.3 ± 30.3 km
Range	--	2.9–65.0 km	3.3–125.5 km
Hourly displacement			
Distance (Mean ± SD)	203 ± 263 m	231 ± 489 m	406 ± 1269 m
Range	0–2069 m	0–10,063 m	0–40,074 m

**Table 3:** Summary of HPAI perpetuation among Swan Geese (*Anser cygnoides*) through the late breeding season from two models (marked bold) that reflect our observed prevalence rate along with similar models sharing initial conditions (2000 agents with 50 infections). Profiles were simulated from 200 iterations.

Wetland Extent	Transmission Rate	Inter-Wetland Movement (%)	Persistence (Days)	Probability of Perpetuation (%)	The Number of Agents at the End of the Late Breeding Season		
					Susceptible	Recovered	Dead
Wet year (2014)	Low (4.4%)	0	61.6 ± 5.6	4.6	932.7 ± 103.4	713.2 ± 70.2	354.1 ± 37.4
		**1**	**65.7 ± 4.8**	**13.0**	**888.5 ± 108.3**	**741.3 ± 74.1**	**370.0 ± 39.0**
		2	67.1 ± 4.4	17.6	855.0 ± 101.5	762.1 ± 70.1	382.5 ± 36.2
	High (9.7%)	0	54.6 ± 7.2	2.6	608.5 ± 104.3	926.6 ± 71.8	464.9 ± 38.9
		1	61.7 ± 5.9	5.8	433.5 ± 94.9	1045.4 ± 67.0	520.9 ± 36.3
		**2**	**63.3 ± 5.7**	**8.0**	**347.5 ± 76.7**	**1101.8 ± 54.3**	**550.6 ± 32.2**
Dry year (2015)	Low (4.4%)	0	62.2 ± 5.9	7.8	510.9 ± 90.0	992.5 ± 63.4	496.5 ± 34.9
		1	66.0 ± 5.1	18.0	449.9 ± 76.2	1032.2 ± 53.5	517.5 ± 31.4
		2	66.8 ± 4.5	16.8	423.7 ± 67.2	1051.6 ± 49.2	524.5 ± 27.9
	High (9.7%)	0	48.6 ± 7.0	0.4	284.6 ± 91.1	1144.5 ± 62.6	570.9 ± 37.1
		1	57.1 ± 7.5	4.8	151.3 ± 58.4	1234.5 ± 42.9	614.1 ± 28.4
		2	56.7 ± 6.6	2.8	112.9 ± 40.1	1258.8 ± 35.3	628.2 ± 24.4

## Data Availability

The data presented in this study are available upon request from the corresponding author or found in the Movebank Data Repository (https://www.movebank.org/cms/movebank-main (accessed on 8 September 2024)).

## Supplementary Materials

The following supporting information can be downloaded at https://www.mdpi.com/article/10.3390/v17020196/s1: Figure S1: SEIR flow model diagram; Figure S2: Comparing telemetry and agent movements; Figure S3: Simulated infection profiles from 1000 agents; Figure S4: Simulated infection profiles from 2000 agents. Text S1: Example parameterization for NetLogo ABM.

## References

[1] World Health Organization (2025). Avian Influenza Weekly Update Number 979.

[2] UNFAO Global Avian Influenza Viruses with Zoonotic Potential Situation Update: Bird Species Affected by H5Nx HPAI. https://www.fao.org/animal-health/situation-updates/global-aiv-with-zoonotic-potential/en.

[3] Takekawa J.Y., Prosser D.J., Newman S.H., Muzaffar S.B., Hill N.J., Yan B., Xiao X., Lei F., Li T., Schwarzbach S.E. (2010). Victims and Vectors: Highly Pathogenic Avian Influenza H5N1 and the Ecology of Wild Birds. Avian Biol. Res..

[4] Nicola M.D., Mocuta D.N. (2021). Strategic Management of the Avian Influentza (AI)/Highly Pathogenic Avian Influenza (HPAI). Sci. Work. Ser. C Vet. Med..

[5] Zhang G., Li B., Raghwani J., Vrancken B., Jia R., Hill S.C., Fournié G., Cheng Y., Yang Q., Wang Y. (2023). Bidirectional Movement of Emerging H5N8 Avian Influenza Viruses between Europe and Asia via Migratory Birds since Early 2020. Soc. Mol. Biol. Evol..

[6] Lee D.H., Bertran K., Kwon J.H., Swayne D.E. (2017). Evolution, Global Spread, and Pathogenicity of Highly Pathogenic Avian Influenza H5Nx Clade 2.3.4.4. J. Vet. Sci..

[7] Caliendo V., Lewis N.S., Pohlmann A., Baillie S.R., Banyard A.C., Beer M., Brown I.H., Fouchier R.A.M., Hansen R.D.E., Lameris T.K. (2022). Transatlantic Spread of Highly Pathogenic Avian Influenza H5N1 by Wild Birds from Europe to North America in 2021. Sci. Rep..

[8] Yoo D.-S., Lee K., Beatriz M.L., Chun B.C., Belkhiria J., Lee K.-N. (2022). Spatiotemporal Risk Assessment for Avian Influenza Outbreak Based on the Dynamics of Habitat Suitability for Wild Birds. Transbound. Emerg. Dis..

[9] Gilbert M., Xiao X., Pfeiffer D.U., Epprecht M., Boles S., Czarnecki C., Chaitaweesub P., Kalpravidh W., Minh P.Q., Otte M.J. (2008). Mapping H5N1 Highly Pathogenic Avian Influenza Risk in Southeast Asia. Proc. Natl. Acad. Sci. USA.

[10] Prosser D.J., Hungerford L.L., Erwin R.M., Ottinger M.A., Takekawa J.Y., Ellis E.C. (2013). Mapping Avian Influenza Transmission Risk at the Interface of Domestic Poultry and Wild Birds. Front. Public Health.

[11] Paul M., Baritaux V., Wongnarkpet S., Poolkhet C., Thanapongtharm W., Roger F., Bonnet P., Ducrot C. (2013). Practices Associated with Highly Pathogenic Avian Influenza Spread in Traditional Poultry Marketing Chains: Social and Economic Perspectives. Acta Trop..

[12] Pinsent A., Pepin K.M., Zhu H., Guan Y., White M.T., Riley S. (2017). The Persistence of Multiple Strains of Avian Influenza in Live Bird Markets. Proc. R. Soc. B Biol. Sci..

[13] Pinotti F., Kohnle L., Lourenço J., Gupta S., Hoque M.A., Mahmud R., Biswas P., Pfeiffer D., Fournié G. (2024). Modelling the Transmission Dynamics of H9N2 Avian Influenza Viruses in a Live Bird Market. Nat. Commun..

[14] Hoye B.J., Munster V.J., Huig N., de Vries P., Oosterbeek K., Tijsen W., Klaassen M., Fouchier R.A.M., van Gils J.A. (2016). Hampered Performance of Migratory Swans: Intra- and Inter-Seasonal Effects of Avian Influenza Virus. Integr. Comp. Biol..

[15] Wang D., Li M., Xiong C., Yan Y., Hu J., Hao M., Liang B., Chen J., Chen G., Yang G. (2021). Ecology of Avian Influenza Viruses in Migratory Birds Wintering within the Yangtze River Wetlands. Sci. Bull..

[16] Lycett S.J., Duchatel F., Digard P. (2019). A Brief History of Bird Flu. Philos. Trans. R. Soc. B Biol. Sci..

[17] Blagodatski A., Trutneva K., Glazova O., Mityaeva O., Shevkova L., Kegeles E., Onyanov N., Fede K., Maznina A., Khavina E. (2021). Avian Influenza in Wild Birds and Poultry: Dissemination Pathways, Monitoring Methods, and Virus Ecology. Pathogens.

[18] Takekawa J.Y., Prosser D.J., Sullivan J.D., Yin S., Wang X., Zhang G., Xiao X. (2023). Potential Effects of Habitat Change on Migratory Bird Movements and Avian Influenza Transmission in the East Asian-Australasian Flyway. Diversity.

[19] Endo A., Nishiura H. (2018). The Role of Migration in Maintaining the Transmission of Avian Influenza in Waterfowl: A Multisite Multispecies Transmission Model along East Asian-Australian Flyway. Can. J. Infect. Dis. Med. Microbiol..

[20] Gilbert M., Jambal L., Karesh W.B., Fine A., Shiilegdamba E., Dulam P., Sodnomdarjaa R., Ganzorig K., Batchuluun D., Tseveenmyadag N. (2012). Highly Pathogenic Avian Influenza Virus among Wild Birds in Mongolia. PLoS ONE.

[21] Lee D.-H., Sharshov K., Swayne D.E., Kurskaya O., Sobolev I., Kabilov M., Alekseev A., Irza V., Shestopaiov A. (2017). Novel Reassortant Clade 2.3.4.4 Avian Influenza A(H5N8) Virus in Wild Aquatic Birds, Russia, 2016. Emerg. Infect. Dis..

[22] van Dijk J.G., Verhagen J.H., Wille M., Waldenström J. (2018). Host and Virus Ecology as Determinants of Influenza A Virus Transmission in Wild Birds. Curr. Opin. Virol..

[23] Fox A.D., Flint P.L., Hohman W.L., Savard J.-P.L. (2014). Waterfowl Habitat Use and Selection during the Remigial Moult Period in the Northern Hemisphere. Wildfowl J..

[24] Tonra C.M., Reudink M.W. (2018). Expanding the Traditional Definition of Molt-Migration. Auk.

[25] Gilbert M., Xiao X., Domenech J., Lubroth J., Martin V., Slingenbergh J. (2006). Anatidae Migration in the Western Palearctic and Spread of Highly Pathogenic Avian Influenza H5N1 Virus. Emerg. Infect. Dis..

[26] Rodríguez-Ochoa A., Kusack J.W., Mugica L., Cruz M.A., Alfonso P., Delgado-Hernández B., Abreu Y., García E., Hobson K.A. (2024). Migratory Connectivity of Blue-Winged Teal: Risk Implications for Avian Influenza Virus Introduction to Cuba. Front. Bird Sci..

[27] Guillemette M., Pelletier D., Grandbois J.-M., Butler P.J. (2007). Flightlessness and the Energetic Cost of Wing Molt in a Large Sea Duck. Ecology.

[28] Weber T.P., Stilianakis N.I. (2007). Ecologic Immunology of Avian Influenza (H5N1) in Migratory Birds. Emerg. Infect. Dis..

[29] Li X., Xu B., Shaman J. (2019). Pathobiological Features Favouring the Intercontinental Dissemination of Highly Pathogenic Avian Influenza Virus. R. Soc. Open Sci..

[30] Macal C.M., North M.J. Agent-Based Modeling and Simulation. Proceedings of the 2009 Winter Simulation Conference.

[31] BirdLife International Anser Cygnoid The IUCN Red List of Threatened Species 2023, *T22679869A228564177*. https://datazone.birdlife.org/species/factsheet/swan-goose-anser-cygnoid/text.

[32] Simonov E., Goroshko O., Tatiana T., Finlayson C., Everard M., Irvine K., McInnes R., Middleton B., van Dam A., Davidson N. (2017). Daurian Steppe Wetlands of the Amur-Heilong River Basin (Russia, China, and Mongolia). The Wetland Book.

[33] Batbayar N., Takekawa J.Y., Newman S.H., Prosser D.J., Natsagdorj T., Xiao X. (2011). Migration Strategies of Swan Geese Anser Cygnoides from Northeast Mongolia. Wildfowl.

[34] Ganbold O., Bing G.-C., Purevee E.G., Jargalsaikhan A., Munkhbayar M., Khujuu T., Paek W.K. (2020). A Note on Avifaunal Community of Khukh Lake Important Bird Area, Eastern Mongolia. Stilt.

[35] Xianghuang L.I., Wang X., Fang L., Batbayar N., Natsagdorj T., Davaasuren B., Damba I., Zhenggang X.U., Cao L., Fox A.D. (2020). Annual Migratory Patterns of Far East Greylag Geese (Anser Anser Rubrirostris) Revealed by GPS Tracking. Integr. Zool..

[36] Yi K., Junjian Z., Batbayar N., Zhenggang X. (2020). Flyway Connectivity and Population Status of the Greylag Goose Anser Anser in East Asia. Wildfowl.

[37] Bliss S. (2020). Mongolia’s Grassland Biomes. Geogr. Bull..

[38] Jia Q., Koyama K., Choi C.Y., Kim H.J., Cao L., Gao D., Liu G., Fox A.D. (2016). Population Estimates and Geographical Distributions of Swans and Geese in East Asia Based on Counts during the Non-Breeding Season. Bird Conserv. Int..

[39] An A., Zhang Y., Cao L., Jia Q., Wang X. (2018). A Potential Distribution Map of Wintering Swan Goose (*Anser cygnoides*) in the Middle and Lower Yangtze River Floodplain, China. Avian Res..

[40] Damba I., Fang L., Yi K., Zhang J., Batbayar N., You J., Moon O.-K., Jin S.D., Liu B., Liu G. (2021). Flyway Structure, Breeding, Migration and Wintering Distributions of the Globally Threatened Swan Goose Anser Cygnoides in East Asia. Wildfowl.

[41] Gilbert M., Xiao X., Robinson T.P. (2017). Intensifying Poultry Production Systems and the Emergence of Avian Influenza in China: A “One Health/Ecohealth” Epitome. Arch. Public Health.

[42] He H., Amat A., Garine-Wichatitsky M., Morand S., Wang C., Vicente J., Vercauteren K.C., Gortazar C. (2021). Characteristics and Perspectives of Disease at the Wildlife-Livestock Interface in Asia. Diseases at the Wildlife—Livestock Interface.

[43] Prosser D.J., Cui P., Takekawa J.Y., Tang M., Hou Y., Collins B.M., Yan B., Hill N.J., Li T., Li Y. (2011). Wild Bird Migration across the Qinghai-Tibetan Plateau: A Transmission Route for Highly Pathogenic H5N1. PLoS ONE.

[44] Choi C., Lee K.-S., Poyarkov N.D., Lee H., Takekawa J.Y., Smith L.M., Ely C.R., Wang X., Cao L., Fox A.D. (2016). Low Survival Rates of Swan Geese (*Anser cygnoides*) Estimated from Neck-Collar Resighting and Telemetry. Waterbirds.

[45] An A., Cao L., Jia Q., Wang X., Zhu Q., Zhang J., Ye X., Gao D. (2019). Changing Abundance and Distribution of the Wintering Swan Goose Anser Cygnoides in the Middle and Lower Yangtze River Floodplain: An Investigation Combining a Field Survey with Satellite Telemetry. Sustainability.

[46] Zhu Q., Hobson K.A., Zhao Q., Zhou Y., Damba I., Batbayar N., Natsagdorj T., Davaasuren B., Antonov A., Guan J. (2020). Migratory Connectivity of Swan Geese Based on Species’ Distribution Models, Feather Stable Isotope Assignment and Satellite Tracking. Divers. Distrib..

[47] Damba I., Zhang J., Yi K., Dou H., Batbayar N., Natsagdorj T., Davaasuren B., Cao L., Fox A.D. (2021). Seasonal and Regional Differences in Migration Patterns and Conservation Status of Swan Geese (*Anser cygnoides*) in the East Asian Flyway. Avian Res..

[48] Xu H. (2006). Modification of Normalised Difference Water Index (NDWI) to Enhance Open Water Features in Remotely Sensed Imagery. Int. J. Remote Sens..

[49] Simonov E.A., Goroshko O., Egidarev E., Kiriliuk O., Kiriliuk V., Kochneva N., Obyazov V., Tkachuk T. (2013). Adaptation to Climate Change in the River Basins of Dauria: Ecology and Water Management.

[50] Kenward R.E., Pfeffer R.H., Al-Bowardi M.A., Fox N.C., Riddle K.E., Bragin E.A., Levin A., Walls S.S., Hodder K.H. (2001). Setting harness sizes and other marking techniques for a falcon with strong sexual dimorphism. J. Field Ornithol..

[51] Kear J. (2005). Ducks, Geese and Swans.

[52] Wilensky U. NetLogo Flocking model. Center for Connected Learning and Computer-Based Modeling, Northwestern University, Evanston, IL, USA, 1998. http://ccl.northwestern.edu/netlogo/models/Flocking.

[53] Wilensky U., Rand W. (2015). Introduction to Agent-Based Modeling: Modeling Natural, Social and Engineered Complex Systems with NetLogo.

[54] Börger L., Franconi N., De Michele G., Gantz A., Meschi F., Manica A., Lovari S., Coulson T. (2006). Effects of Sampling Regime on the Mean and Variance of Home Range Size Estimates. J. Anim. Ecol..

[55] Gitzen R.A., Millspaugh J.J., Kernohan B.J. (2006). Bandwidth Selection for Fixed-Kernel Analysis of Animal Utilization Distributions. J. Wildl. Manag..

[56] Hemson G., Johnson P., South A., Kenward R., Ripley R., Mcdonald D. (2005). Are Kernels the Mustard? Data from Global Positioning System (GPS) Collars Suggests Problems for Kernel Home-Range Analyses with Least-Squares Cross-Validation. J. Anim. Ecol..

[57] Duong T. Ks: Kernel Density Estimation for Bivariate Data. https://cran.r-project.org/web/packages/ks/vignettes/kde.pdf.

[58] Millspaugh J.J., Gitzen R.A., Kernohan B.J., Larson M.A., Clay C.L. (2004). Comparability of Three Analytical Techniques to Assess Joint Space Use. Wildl. Soc. Bull..

[59] Long J.A., Nelson T.A., Webb S.L., Gee K.L. (2014). A Critical Examination of Indices of Dynamic Interaction for Wildlife Telemetry Studies. J. Anim. Ecol..

[60] Chen Y., Liu F., Yu Q., Li T. (2021). Review of Fractional Epidemic Models. Appl. Math. Model..

[61] Gharakhanlou N., Hooshangi N. (2020). Spatio-Temporal Simulation of the Novel Coronavirus (COVID-19) Outbreak Using the Agent-Based Modeling Approach (Case Study: Urmia, Iran). Inform. Med. Unlocked.

[62] Longini I.M., Koopman J.S. (1982). Household and Community Transmission Parameters from Final Distributions of Infections in Households. Biometrics.

[63] Hope-Simpson R., Sutherland I. (1954). Does Influenza Spread within the Household. Lancet.

[64] Brown J.D., Stallknecht D.E., Swayne D.E. (2008). Experimental Infection of Swans and Geese with Highly Pathogenic Avian Influenza Virus (H5N1) of Asian Lineage. Emerg. Infect. Dis..

[65] Gaidet N., Cappelle J., Takekawa J.Y., Prosser D.J., Iverson S.A., Douglas D.C., Perry W.M., Mundkur T., Newman S.H. (2010). Potential Spread of Highly Pathogenic Avian Influenza H5N1 by Wildfowl: Dispersal Ranges and Rates Determined from Large-Scale Satellite Telemetry. J. Appl. Ecol..

[66] Wearing H.J., Rohani P., Keeling M.J. (2005). Appropriate Models for the Management of Infectious Diseases. PLoS Med..

[67] Prosser D.J., Schley H.L., Simmons N., Sullivan J.D., Homyack J., Weegman M., Olsen G.H., Berlin A.M., Poulson R.L., Stallknecht D.E. (2022). A Lesser Scaup (*Aythya affinis*) Naturally Infected with Eurasian 2.3.4.4 Highly Pathogenic H5N1 Avian Influenza Virus: Movement Ecology and Host Factors. Transbound. Emerg. Dis..

[68] Teitelbaum C.S., Casazza M.L., McDuie F., De La Cruz S.E.W., Overton C.T., Hall L.A., Matchett E.L., Ackerman J.T., Sullivan J.D., Ramey A.M. (2023). Waterfowl Recently Infected with Low Pathogenic Avian Influenza Exhibit Reduced Local Movement and Delayed Migration. Ecosphere.

[69] Samuel M.D., Hall J.S., Brown J.D., Goldberg D.R., Ip H., Baranyuk V.V. (2015). The Dynamics of Avian Influenza in Lesser Snow Geese: Implications for Annual and Migratory Infection Patterns. Ecol. Appl..

[70] Brown J.D., Stallknecht D.E., Beck J.R., Suarez D.L., Swayne D.E. (2006). Susceptibility of North American Ducks and Gulls to H5N1 Highly Pathogenic Avian Influenza Viruses. Emerg. Infect. Dis..

[71] Germeraad E.A., Sanders P., Hagenaars T.J., De Jong M.C.M., Beerens N., Gonzales J.L. (2019). Virus Shedding of Avian Influenza in Poultry: A Systematic Review and Meta-Analysis. Viruses.

[72] Löndt B.Z., Núñez A., Banks J., Alexander D.J., Russell C., Richard- Löndt A.C., Brown I.H. (2010). The Effect of Age on the Pathogenesis of a Highly Pathogenic Avian Influenza (HPAI) H5N1 Virus in Pekin Ducks (*Anas Platyrhynchos*) Infected Experimentally. Influenza Other Respir. Viruses.

[73] Pantin-Jackwood M.J., Costa-Hurtado M., Shepherd E., DeJesus E., Smith D., Spackman E., Kapczynski D.R., Suarez D.L., Stallknecht D.E., Swayne D.E. (2016). Pathogenicity and Transmission of H5 and H7 Highly Pathogenic Avian Influenza Viruses in Mallards. J. Virol..

[74] Berhane Y., Embury-Hyatt C., Leith M., Kehler H., Suderman M., Pasick J. (2014). Pre-Exposing Canada Geese (*Branta canadensis*) to a Low-Pathogenic H1N1 Avian Influenza Virus Protects Them against H5N1 HPAI Virus Challenge. J. Wildl. Dis..

[75] Tarasiuk K., Kycko A., Świętoń E., Bocian Ł., Wyrostek K., Śmietanka K. (2023). Homo- and Heterosubtypic Immunity to Low Pathogenic Avian Influenza Virus Mitigates the Clinical Outcome of Infection with Highly Pathogenic Avian Influenza H5N8 Clade 2.3.4.4.b in Captive Mallards (*Anas platyrhynchos*). Pathogens.

[76] R Development Core Team (2012). R: A Language and Environment for Statistical Computing.

[77] Hegemann A., Birberg C., Hasselquist D., Nilsson J. (2022). Early and Late Migrating Avian Individuals Differ in Constitutive Immune Functions and Blood Parasite Infections—But Patterns Depend on Migratory Strategy. Front. Ecol. Evol..

[78] Chen R., Holmes E.C. (2009). Frequent Inter-Species Transmission and Geographic Subdivision in Avian Influenza Viruses from Wild Birds. Virology.

[79] RAMSAR (1997). Ramsar Information Sheet—Mongol Daguur.

[80] Yin S., Xu C., Zhang Y., de Boer W.F., Mundkur T., Artois J., Velkers F.C., Takekawa J.Y., Si Y., Tian H. (2024). Strong and Consistent Effects of Waterbird Composition on HPAI H5 Occurrences across Europe. Ecol. Appl..

[81] Yin S., Xu Y., de Jong M.C.M., Huisman M.R.S., Contina A., Prins H.H.T., Huang Z.Y.X., de Boer W. (2021). Habitat Loss Exacerbates Pathogen Spread: An Agent-Based Model of Avian Influenza Infection in Migratory Waterfowl. PLoS Comp. Biol..

[82] Pederson N., Hessl A.E., Baatarbileg N., Anchukaitis K.J., Di Cosmo N. (2014). Pluvials, Droughts, the Mongol Empire, and Modern Mongolia. Proc. Natl. Acad. Sci. USA.

[83] An Q., He H., Nie Q., Cui Y., Gao J., Wei C., Xie X., You J. (2020). Spatial and Temporal Variations of Drought in Inner Mongolia, China. Water.

[84] Wen Y., Wang X., Liu H., Yu H., Niu F., Fang H., Wen L., Zhuo Y., Fu R., Bai Y. (2024). Dynamic Evolution and Driving Forces of the Changes in Lakeside Wetlands in the Mongolian Plateau. J. Resour. Ecol..

[85] Han J., Dai H., Gu Z. (2021). Sandstorms and Desertification in Mongolia, an Example of Future Climate Events: A Review. Environ. Chem. Lett..

[86] Nandintsetseg B., Boldgiv B., Chang J., Ciais P., Davaanyam E., Batbold A., Bat-Oyun T., Stenseth N.C. (2021). Risk and Vulnerability of Mongolian Grasslands under Climate Change. Environ. Res. Lett..

[87] Boykova E., Campi A.J., Panda J. (2021). Environmental Security Issues in Northeast Asia and Cooperation among Russia, China, and Mongolia. Mongolia and Northeast Asian Security.

[88] Bai M., Mo X., Liu S., Hu S. (2021). Detection and Attribution of Lake Water Loss in the Semi-Arid Mongolian Plateau—A Case Study in the Lake Dalinor. Ecohydrology.

[89] Kramer L.D., Ciota A.T., Marm Kilpatrick A. (2019). Introduction, Spread, and Establishment of West Nile Virus in the Americas. J. Med. Entomol..

