# Supplementary material for: Perpetuation of Avian Influenza from Molt to Fall Migration in Wild Swan Geese (Anser cygnoides): An Agent-Based Modeling Approach
Source: Viruses. 2025 Jan 30;17(2):196. doi: 10.3390/v17020196 (PMC11861497; doi:10.3390/v17020196)
Supplement: Supplementary file 1 [file viruses-17-00196-s001.zip › viruses-3257026-supplementary.pdf]

Supplementary Figures for:

Perpetuation of Avian Influenza from Molt to Fall Migration in Wild Swan Geese (*Anser cygnoides*): An Agent-Based Modeling Approach

John Y. Takekawa [1,2,*,†], Chang-Yong Choi [1,2,3], Diann J. Prosser [4], Jeffery D. Sullivan [4], Nyambayar Batbayar [5] and Xiangming Xiao [2]

[1] U.S. Geological Survey, Western Ecological Research Center, Vallejo, CA 94592, USA
[2] School of Biological Sciences, University of Oklahoma, Norman, OK 73019, USA
[3] Department of Forest Sciences, Seoul National University, Seoul 08826, Republic of Korea
[4] U.S. Geological Survey, Eastern Ecological Science Center, Laurel, MD 20708, USA
[5] Wildlife Science and Conservation Center, Ulaanbaatar 210351, Mongolia
* Correspondence: jtakekawa@suisunrcd.org
† Current address: Suisun Resource Conservation District, Suisun City, CA 94585, USA.

Any use of trade, firm, or product names is for descriptive purposes only and does not imply endorsement by the U.S. Government.

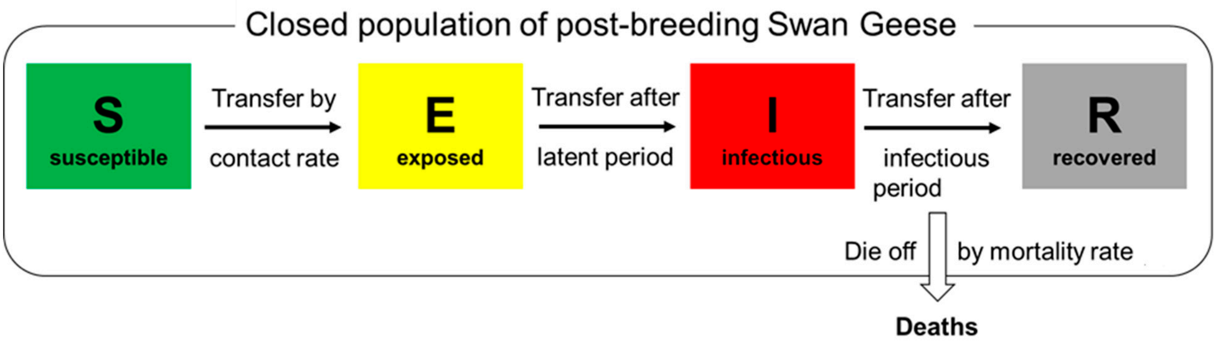

Figure S1. Flow diagram of the infection states of an agent based on susceptible-exposed-infectious-recovered (SEIR) model.

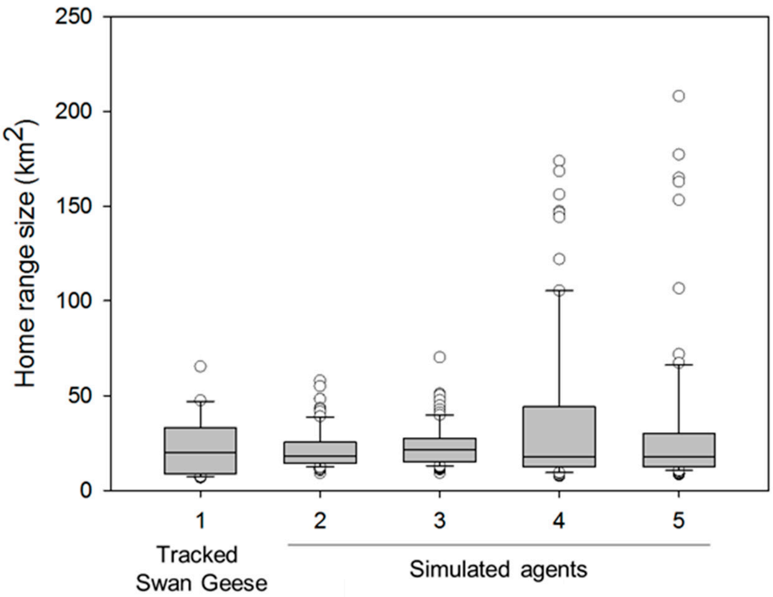

Figure S2. Comparison of 90% kernel home ranges calculated from the telemetry data of Swan Geese marked at four molting lakes (Group 1; n=37) and the simulated agents in the agent-based model (Group 2-5; n=80 for each group). Agents in the Group 2 and 3 were initially placed at the same lakes where the tracked geese were marked, whereas Group 4 and 5 were randomly distributed agents. Simulations were independently performed 20 times under 1,000 (Group 2 and 4) and 2,000 agent environments (Group 3 and 5).

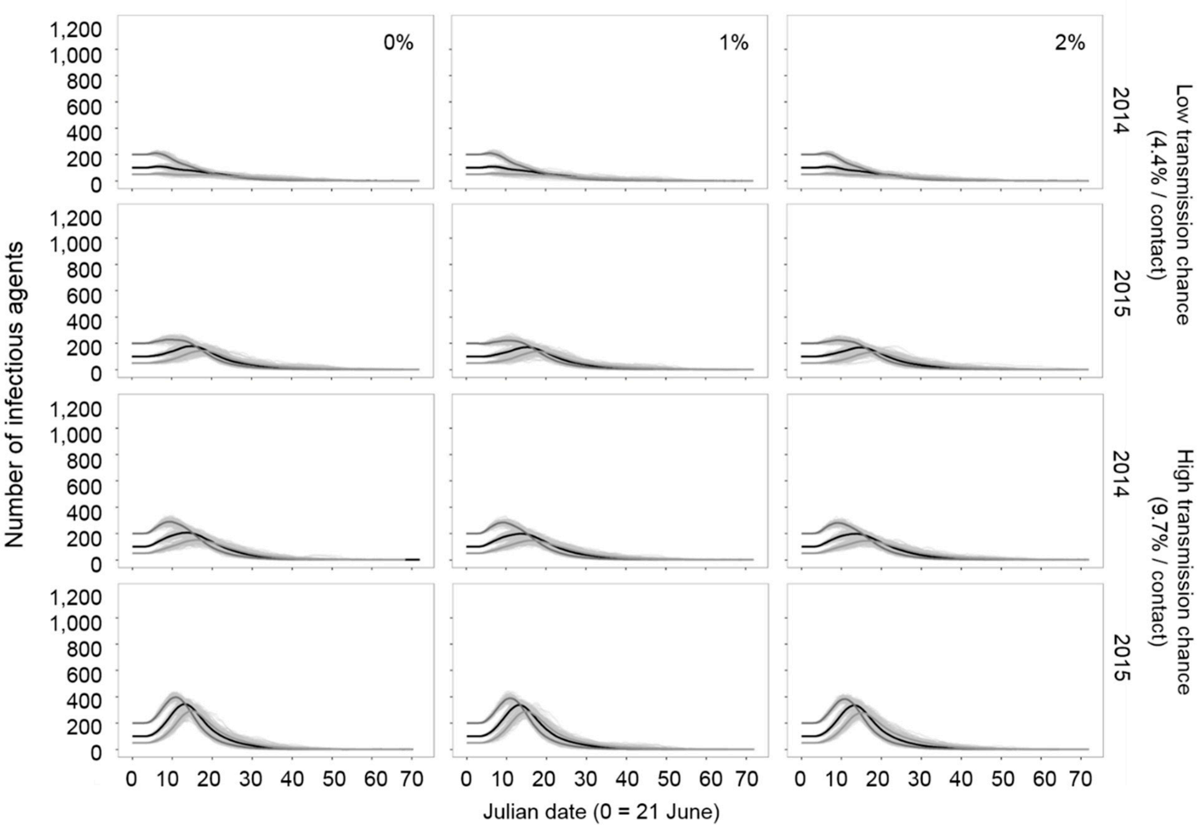

Figure S3. Infection profiles of HPAI in the Swan Goose (*Anser cygnoides*) simulated from 200 iterations using 1,000 agents in 2014 (wet year) and 2015 (dry year). Lines indicate the mean number of infectious agents starting from 50, 100, and 200 initial infections, respectively. Percentage in top right of each column means the daily probability of inter-wetland movement in the molting stage (left column: 0%, center: 1%, right: 2%).

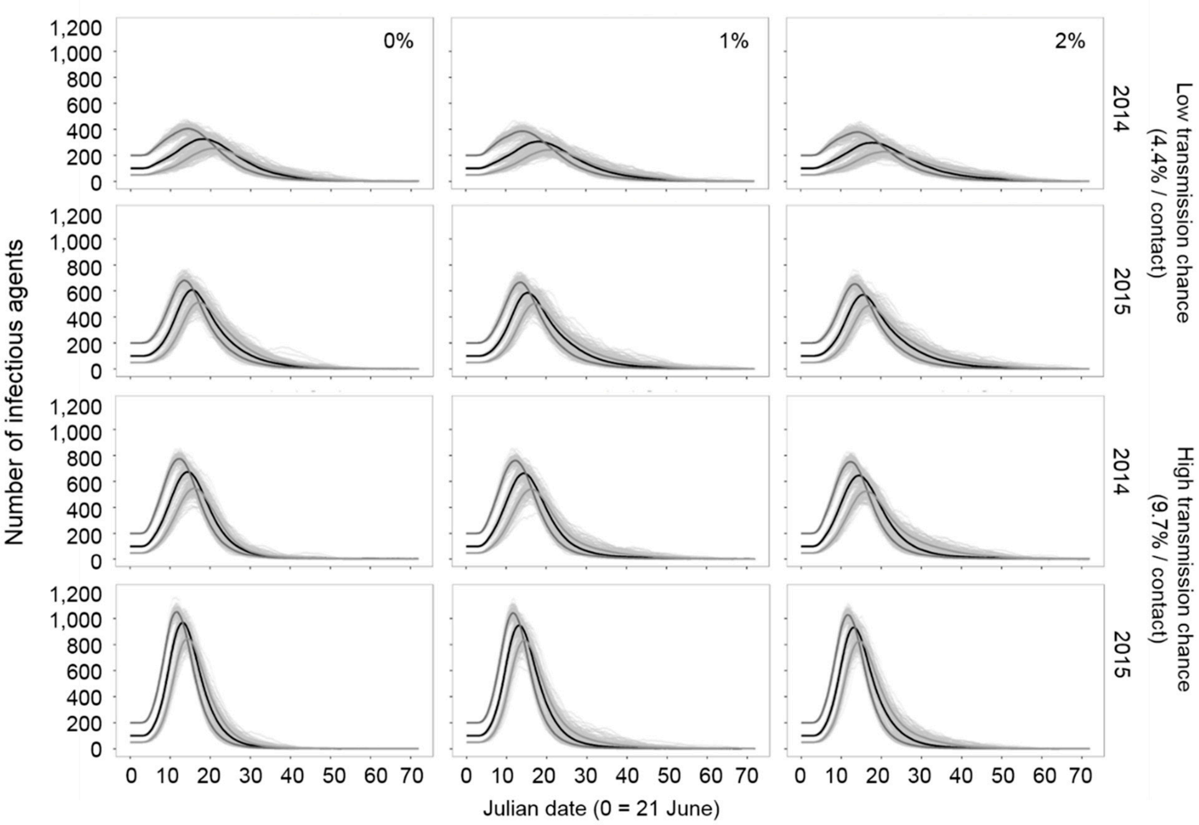

Figure S4. Infection profiles of HPAI in the Swan Goose (*Anser cygnoides*) simulated from 200 iterations using 2,000 agents in 2014 (wet year) and 2015 (dry year). Lines indicate the mean number of infectious agents starting from 50, 100, and 200 initial infections, respectively. Percentage in top right of each column means the daily probability of inter-wetland movement in the molting stage (left column: 0%, center: 1%, right: 2%). Lines indicate the mean number of infectious agents starting from 50, 100, and 200 initial infections, respectively. Percentage in top right of each column means the daily probability of inter-wetland movement in the molting stage (left column: 0%, center: 1%, right: 2%).

Supplementary Text S1. Example parameterization for NetLogo ABM. This text describes the basic parameterizations that can be used to recreate these results within NetLogo. However, this does not explicitly serve as recreated code as variables must be entered manually within the program as outlined within Table 1 of "Perpetuation of avian influenza from molt to fall migration in wild Swan Geese (Anser cygnoides): an agent-based modeling approach".

```
extensions [ gis ]          ;; Activate GIS extentions

globals
[ elevation                 ;; Gradients (distance) to selected molting lakes
  moltattract               ;; Gradients (distance) to available wetlands
  site                      ;; Study area raster
  width                     ;; Size of the raster (width in pixels)
  height                    ;; Size of the raster (height in pixels)
  time                      ;; Time and date of a simulation
  time1                     ;; Time and date of a simulation
  time2                     ;; Time and date of a simulation
  no_infect_sampling        ;; Positive/infectious birds at the time of sampling (day 36: 27 July 2014: 864
tick)
  maximum-infectious        ;; Maximum number of infectious geese
  tick-at-maximum-infectious   ;; Time of the maximum number of infectious individuals
  number-infectious-bird    ;; Initial number of infectious geese
  incubation-alpha          ;; Shape (alpha) parameter for the gamma distribution of incubation period
  incubation-lambda         ;; Scale (lambda) parameter for the gamma distribution of incubation period
  infectious-alpha          ;; Shape (alpha) parameter for the gamma distribution of infection period
  infectious-lambda         ;; Scale (lambda) parameter for the gamma distribution of infection period
  ]

turtles-own
[
  total-contacts            ;; A count of all contacts of the individual
  flockmates                ;; Agentset of nearby turtles found in a given radius
  nearest-neighbor          ;; Nearest/closest bird among found flockmates

  susceptible?              ;; True indicates that the agent (bird) is in the susceptible class.
```

exposed?   ;; True indicates that the agent (bird) is in the exposed class (incubating the HPAI virus).

infectious?   ;; True indicates that the agent (bird) is in the infectious class.

recovered?   ;; True indicates that the agent (bird) is in the recovered class.

stationed?   ;; True indicates that the agent (bird) is in wetlands.

record?   ;; Temporary class status for recording locations for homerange analysis

incubation-length   ;; Time lapse of virus incubation in each exposed individual

incubation-time   ;; Randomly selected time of transition from exposed to infectious classes (following gamma-distribution)

infectious-length   ;; Time lapse of virus shedding in each infectious individual

infectious-time   ;; Randomly selected time of transition from infectious to recovered/dead classes (following gamma-distribution)

]

;;
;;;;;;;;;;;;;;;;;;;;;;;;;;

;;;;;;;;;;;;;;;;;;;;;; General setup ;;;

;;;
;;;;;;;;;;;;;;;;;;;;;;;;;;

to setup

clear-all

set site gis:load-dataset "data/basemap.asc"

gis:set-world-envelope gis:envelope-of site   ;; Set extent

gis:paint site 0

set moltattract gis:load-dataset "data/distance_major.asc"   ;; Set gradients of elevational difference for initial migration to 41 selected molting lakes

set elevation gis:load-dataset "data/distance_all.asc"   ;; Set gradients of elevational difference for general movements to available wetlands

set width gis:width-of elevation   ;; Set extent size

set height gis:height-of elevation   ;; Set extent size

gis:set-world-envelope gis:envelope-of elevation

reset-ticks

setup-gamma-distribution

setup-population

set time date-and-time

set time1 replace-item 2 time "-"

set time2 replace-item 5 time1 "-"

;; output-type "Year" output-type "," output-type "sim_id" output-type "," output-type "ticks" output-type "," output-type "infectious" output-type "," output-type "exposed" output-type "," output-type "susceptible" output-type "," output-type "recovered" output-type ","

;; output-type "dead" output-type ","   output-type "alive" output-type "," output-type "max-no-infectious" output-type "," output-type "max-day-infectious" output-type "," output-type "no-in-wetlands" output-type ","

;; output-type "### Model Parameters," output-type "initial-swgo," output-type initial-swgo output-type ", no-HPAI," output-type no-HPAI output-type ", Contact_raidus," output-type contact_radius output-type ", Mortality," output-type mortality output-type ", Transmission_chance," output-print Transmission_chance

end

;;

;;;;;;;;;;;;; Gamma distribution of latent and infectious periods ;;

;;

;;;;;;;;;;;;; For gamma: alpha = mean * mean / variance; lambda = 1 / (variance / mean) ;;;

;;;

to setup-gamma-distribution ;; Transforming the mean and standard deviation for the gamma distribution (alpha as shape parameter and lambda as scale parameter)

set incubation-alpha (average-incubation-period * ticks-per-day)^ 2 / (incubation-standard-deviation * ticks-per-day)^ 2 ;; shape parameter

set incubation-lambda (average-incubation-period * ticks-per-day) / (incubation-standard-deviation * ticks-per-day)^ 2 ;; scale parameter

set infectious-alpha (average-infectious-period * ticks-per-day)^ 2 / (infectious-standard-deviation * ticks-per-day)^ 2 ;; shape parameter

set infectious-lambda (average-infectious-period * ticks-per-day) / (infectious-standard-deviation * ticks-per-day)^ 2 ;; scale parameter

```
end

;;;;;;;;;;;;;;;;;;;;;;;;;;;;;;;;;;;;;;;;;;;;;;;;;;;;;;;;;;;;;;;;;;;;;;;;;;;;;;;;;;;;;;;;;;;;;;;;;;;;;;;;;;;;;;;;;;;;;;;;;;;;;;;;;;;;;;;;;;;;;;;;;;;;;;;;;;;;;;;;;;;;;;;;;;;;;;;;;;;;
;;;;;;;;

;;;;;;;;;;;;;; Population setup
;;;;;;;;;;;;;;;;;;;;;;;;;;;;;;;;;;;;;;;;;;;;;;;;;;;;;;;;;;;;;;;;;;;;;;;;;;;;;;;;;;;;;;;;;;;;;;;;;;;;;;;;;;;;;;;;;;;;;;;;;;;;;;;;;;;;;;;;;;;;;;;;;;;;;;;;;;;;;;;

;;;;;;;;;;;;;;;;;;;;;;;;;;;;;;;;;;;;;;;;;;;;;;;;;;;;;;;;;;;;;;;;;;;;;;;;;;;;;;;;;;;;;;;;;;;;;;;;;;;;;;;;;;;;;;;;;;;;;;;;;;;;;;;;;;;;;;;;;;;;;;;;;;;;;;;;;;;;;;;;;;;;;;;;;;;;;;;;;;;;;
;;;;;;;;

to setup-population
 create-turtles initial-swgo
 [ setxy random-xcor random-ycor       ;; Random distribution of SWGOs in the arena
   set susceptible? true               ;; SWGOs are susceptible agents at the begining of a simulation.
   set exposed? false                  ;; SWGOs do not incubate HPAI at the begining of a simulation.
   set infectious? false               ;; SWGOs do not shed HPAI virus at the begining of a simulation.
   set recovered? false                ;; SWGOs are not immune to HPAI virus at the begining of a simulation.
   set total-contacts 0
   set size 5
   set record? false                   ;; Setting to record location fixes
   set stationed? false                ;; Setting to count agents in wetlands



   ask turtle 1                        ;; Individual 0 begins as infectious.  Its infectious-time is selected from the gamma distribution and infectious-length set to 0.
   [
     setxy 749 723                     ;; Gallut lake
     set susceptible? false
     set infectious? false
     set infectious-time random-gamma infectious-alpha infectious-lambda
     set infectious-length 0
     set record? true
     set color 1
   ]
```

```
ask turtle 2                    ;; Individual 0 begins as infectious.  Its infectious-time is selected from the gamma distribution and infectious-length set to 0.
  [
    setxy 657 727               ;; Bus lake
    set susceptible? false
    set infectious? false
    set infectious-time random-gamma infectious-alpha infectious-lambda
    set infectious-length 0
    set record? true
    set color 2
  ]

ask turtle 3                    ;; Individual 0 begins as infectious.  Its infectious-time is selected from the gamma distribution and infectious-length set to 0.
  [
    setxy 316 676               ;; Kaichilin lake
    set susceptible? false
    set infectious? false
    set infectious-time random-gamma infectious-alpha infectious-lambda
    set infectious-length 0
    set record? true
    set color 3
      ]

ask turtle 4                    ;; Individual 0 begins as infectious.  Its infectious-time is selected from the gamma distribution and infectious-length set to 0.
  [
    setxy 299 505               ;; Ch lake
    set susceptible? false
    set infectious? false
    set infectious-time random-gamma infectious-alpha infectious-lambda
    set infectious-length 0
```

```
    set record? true
       set color 4
  ]

  ask turtle 5              ;; Individual 0 begins as infectious.  Its infectious-time is selected from the gamma distribution and infectious-length set to 0.
  [
    setxy random-xcor random-ycor              ;; Gallut lake
    set susceptible? false
    set infectious? false
    set infectious-time random-gamma infectious-alpha infectious-lambda
    set infectious-length 0
    set record? true
    set color 5
  ]

  ask turtle 6              ;; Individual 0 begins as infectious.  Its infectious-time is selected from the gamma distribution and infectious-length set to 0.
  [
    setxy random-xcor random-ycor              ;; Bus lake
    set susceptible? false
    set infectious? false
    set infectious-time random-gamma infectious-alpha infectious-lambda
    set infectious-length 0
    set record? true
    set color 6
  ]

  ask turtle 7              ;; Individual 0 begins as infectious.  Its infectious-time is selected from the gamma distribution and infectious-length set to 0.
  [
    setxy random-xcor random-ycor              ;; Kaichilin lake
```

```
    set susceptible? false
    set infectious? false
    set infectious-time random-gamma infectious-alpha infectious-lambda
    set infectious-length 0
    set record? true
    set color 7
      ]

  ask turtle 8                  ;; Individual 0 begins as infectious.  Its infectious-time is selected from the gamma distribution and infectious-length set to 0.
  [
    setxy random-xcor random-ycor              ;; Ch lake
    set susceptible? false
    set infectious? false
    set infectious-time random-gamma infectious-alpha infectious-lambda
    set infectious-length 0
    set record? true
    set color 8
  ]

  if who < no-HPAI              ;; Create infectious birds as given number of no-HPAI (with unknown time lapse of being infectious)
    [repeat no-HPAI
      [set susceptible? false
       set infectious? true
       set infectious-time random-gamma infectious-alpha infectious-lambda
       set infectious-length random (average-infectious-period * -1 * ticks-per-day)          ;; The bird is currently infectious at the start of simulation, while the timing of status change is unknown but within the average-infectious-period.
       set record? false ]]

  set number-infectious-bird [ 0 ]                                    ;; The number-infectious-bird vector is initiallized.
```

```
    assign-color
  ]

end

;;;;;;;;;;;;;;;;;;;;;;;;;;;;;;;;;;;;;;;;;;;;;;;;;;;;;;;;;;;;;;;;;;;;;;;;;;;;;;;;;;;;;;;;;;;;;;;;;;;;;;;;;;;;;;;;;;;;;;;;;;;;;;;;;;;;;;;
;;;;;;;;;;;;; Color of birds by status ;;;;;;;;;;;;;;;;;;;;;;;;;;;;;;;;;;;;;;;;;;;;;;;;;;;;;;;;;;;;;;;;;;;;;;;;;;;;;;;;;;;;;;;;;;;;;;
;;;;;;;;;;;;;;;;;;;;;;;;;;;;;;;;;;;;;;;;;;;;;;;;;;;;;;;;;;;;;;;;;;;;;;;;;;;;;;;;;;;;;;;;;;;;;;;;;;;;;;;;;;;;;;;;;;;;;;;;;;;;;;;;;;;;;;;

to assign-color
  if susceptible?
    [ set color green ]
  if exposed?
    [ set color yellow ]
  if infectious?
    [ set color red ]
  if recovered?
    [ set color grey ]
end

;;;;;;;;;;;;;;;;;;;;;;;;;;;;;;;;;;;;;;;;;;;;;;;;;;;;;;;;;;;;;;;;;;;;;;;;;;;;;;;;;;;;;;;;;;;;;;;;;;;;;;;;;;;;;;;;;;;;;;;;;;;;;;;;;;;;;;;
;;;;;;;;;;;;;;;;;;; Go and data output ;;;;;;;;;;;;;;;;;;;;;;;;;;;;;;;;;;;;;;;;;;;;;;;;;;;;;;;;;;;;;;;;;;;;;;;;;;;;;;;;;;;;;;;;;;;;;;;
;;;;;;;;;;;;;;;;;;;;;;;;;;;;;;;;;;;;;;;;;;;;;;;;;;;;;;;;;;;;;;;;;;;;;;;;;;;;;;;;;;;;;;;;;;;;;;;;;;;;;;;;;;;;;;;;;;;;;;;;;;;;;;;;;;;;;;;

to go

  ask turtles with [ record? ]                              ;; Sample collection for HPAI surveillance in the field in 2014 (day 42: 1 Aug 2014 ; 24*42 = 1008)
    [if ticks >= 1008
  ;;   [file-write ticks file-write "," file-write color file-write "," file-write xcor file-write "," file-write ycor file-write ";"]]
     [output-type ticks output-type "," output-type color output-type "," output-type  xcor * 100 output-type "," output-print ycor * 100]]
```

```
 ifelse ticks >= 1752                              ;; Stop this simulation at the end of the study days: 73 days (1752 hours)
;;[stop]                                     ;; Codes for a single run
  [export-progress
    export-final
    setup
    go]                                      ;; Codes for multiple runs

  [export-progress

  if all? turtles [ susceptible? or recovered? ]               ;; If no individuals are exposed or infectious, stop the simulation.
;; [export-final  stop]                              ;; Codes for a single run
   [export-final
    setup
    go]                                      ;; Codes for multiple runs

  if not any? turtles                                  ;; If there is no live/survived/recovered goose, stop the simulation.
;; [stop]                                    ;; Codes for a single run
   [export-progress
    export-final
    setup
    go]                                      ;; Codes for multiple runs

  if ticks < 1097                                  ;; Typical and local movements of swan geese at the first stage (no interwetland movement) until 45.7 days (1096.8 hours or ticks)
    [ask turtles [molt]]

  if ticks >= 1097                                  ;; Start of the second stage: 45.7 days (1096.8 hours or ticks)
    [ask turtles [move]]

  if ticks > 1509.6                                 ;; Start of the third stage: 62.9 days (1509.6 hours or ticks)
    [ask turtles [premigrate]]
```

```
  if ticks = 936                                   ;; Sample collection for HPAI surveillance in the field in
2014 (day 39: 29 July 2014 ; 24*39 = 936)

    [set no_infect_sampling count turtles with [infectious?]]



  ask turtles with [ exposed? ]                        ;; Exposed geese will become infectious geese after the
incubation-time.

    [ chance-of-becoming-infectious ]


  ask turtles with [ infectious? ]                     ;; Infectious geese may transmit HPAI virus to
susceptible neighbors during the infectious-time and may recover from infection after that time.

    [ expose-neighbors

      chance-of-recovery ]


  ask turtles

    [ assign-color

      count-contacts ]


  ask turtles [flock]


  compute-maximum-infectious


  tick

  ]


end



;;;;;;;;;;;;;;;;;;;;;;;;;;;;;;;;;;;;;;;;;;;;;;;;;;;;;;;;;;;;;;;;;;;;;;;;;;;;;;;;;;;;;;;;;;;;;;;;;;;;;;;;;;;;;;;;;;;;;;;;;;;;;;;;;;;;;;;;;;;;;;;;;;;;;;;;;;;;;;;;;;;
;;;

;;;;;;;;;;;;;;;; Display simulation data
;;;;;;;;;;;;;;;;;;;;;;;;;;;;;;;;;;;;;;;;;;;;;;;;;;;;;;;;;;;;;;;;;;;;;;;;;;;;;;;;;;;;;;;;;;;;;;;;;;;;;;;;;;;;;;;;;;;;;;;;;;

;;;;;;;;;;;;;;;;;;;;;;;;;;;;;;;;;;;;;;;;;;;;;;;;;;;;;;;;;;;;;;;;;;;;;;;;;;;;;;;;;;;;;;;;;;;;;;;;;;;;;;;;;;;;;;;;;;;;;;;;;;;;;;;;;;;;;;;;;;;;;;;;;;;;;;;;;;;;;;;;;;;;;;
;;;
```

```
to export-progress

;; Outputs (year, ticks, infectious, exposed, susceptible, recovered, dead, alive, maximum-infectious, day-max-inf, attack_rate, no-in-wetlands)

;;   output-type "2014" output-type "," output-type substring time2 0 12 output-type "," output-type ticks output-type "," output-type count turtles with [infectious?] output-type "," output-type count turtles with [exposed?] output-type ","

;;   output-type count turtles with [susceptible?] output-type "," output-type count turtles with [recovered?] output-type "," output-type (initial-swgo - count turtles) output-type ","  output-type count turtles output-type ","

;;   output-type maximum-infectious output-type ","  output-type precision (tick-at-maximum-infectious / ticks-per-day) 2 output-type ","  output-print count turtles with [ stationed? ]

end



;;;;;;;;;;;;;;;;;;;;;;;;;;;;;;;;;;;;;;;;;;;;;;;;;;;;;;;;;;;;;;;;;;;;;;;;;;;;;;;;;;;;;;;;;;;;;;;;;;;;;;;;;;;;;;;;;;;;;;;;;;;;;;;;;;;;;;;;;;;;;;;;;;;;;;;;;;;;;;;;;;;;;;;;;;;;;;;;;;;;;;;;;;;;;;;;;;;;;;;;;;;;;;;;;;;;;;;;;

;;;;;;;;;;;;;;;; Export final results ;;;;;;;;;;;;;;;;;;;;;;;;;;;;;;;;;;;;;;;;;;;;;;;;;;;;;;;;;;;;;;;;;;;;;;;;;;;;;;;;;;;;;;;;;;;;;;;;;;;;;;;;;;;;;;;;;;;;;;;;;;;;;;;;;;;;;;;;;;;;

;;;;;;;;;;;;;;;;;;;;;;;;;;;;;;;;;;;;;;;;;;;;;;;;;;;;;;;;;;;;;;;;;;;;;;;;;;;;;;;;;;;;;;;;;;;;;;;;;;;;;;;;;;;;;;;;;;;;;;;;;;;;;;;;;;;;;;;;;;;;;;;;;;;;;;;;;;;;;;;;;;;;;;;;;;;;;;;;;;;;;;;;;;;;;;;;;;;;;;;;;;;;;;;;;;;;;;;;;;;;


to export-final

   export-output (word "c:/Users/subbuteo/Desktop/ABM for migration/output/homerange/hr_2014_"(initial-swgo)"_"(no-HPAI)"_"(average-infectious-period)"_"(Transmission_chance)"_"(wetland-changeI)"_"(substring time2 6 12) ".csv")

   clear-output

;;    output-print "year, sim_id, initial_swgo, no-HPAI, average-infectious-period, transmission_chance, wetland-changeI, persistence, susceptible, exposed, infectious, recovered, dead, %_dead, peak-no-infec, peak-day-infec, no_infect_sampling, %_infect_sampling, attack-rate, mean-daily-contact"

;;    output-type "2014" output-type "," output-type substring time2 0 12 output-type "," output-type initial-swgo output-type "," output-type no-HPAI output-type "," output-type average-infectious-period output-type "," output-type Transmission_chance output-type "," output-type wetland-changeI output-type ","

;;    output-type (ticks / 24) output-type "," output-type count turtles with [susceptible?] output-type "," output-type count turtles with [exposed?] output-type "," output-type count turtles with [infectious?] output-type "," output-type count turtles with [recovered?] output-type "," output-type (initial-swgo - count turtles) output-type "," output-type ((initial-swgo - count turtles) * 100 / initial-swgo)

;;    output-type "," output-type maximum-infectious output-type "," output-type precision (tick-at-maximum-infectious / ticks-per-day) 2  output-type "," output-type no_infect_sampling output-type ","
```

output-type (no_infect_sampling * 100) / count turtles output-type "," output-type (count turtles with [recovered?] * 100 / initial-swgo) output-type ","

;;   output-print (mean [total-contacts / (ticks / ticks-per-day)] of turtles)

;;   export-output (word "c:/Users/subbuteo/Desktop/ABM for migration/output/2014/f_2014_" (initial-swgo)"_"(no-HPAI)"_"(average-infectious-period)"_"(Transmission_chance)"_"(wetland-changeI)"_"(substring time2 6 12) ".csv")

end

;;

;;;;;;;;;;;; SIER model
;;;

;;

to count-contacts

  set total-contacts total-contacts + count other turtles in-radius (contact_radius / 100)   ;; Definition of contact ==> No. of individuals within a contact radius (scale: 100m)

end

to expose-neighbors

  ask other turtles in-radius (contact_radius / 100) with [susceptible?]   ;; Susceptible birds that contacted with an infectious goose get infected with the preset probability of transmission.

    [

     if random-float 100 < random-normal Transmission_chance Transmission_chance_deviation

       [set susceptible? false

         set exposed? true   ;; Newly exposed birds chage their status from susceptibel to exposed.

         set incubation-time random-gamma incubation-alpha incubation-lambda   ;; Newly exposed birds randomly set incubation-time following the gamma distribution with the given parameters.

         set incubation-length 0   ;; Newly exposed birds start their incubation-lenth of 0.

       ]

```
  ]
end

to chance-of-becoming-infectious                                    ;; When an infected individual has been in the exposed class longer than its incubation-time, it will become infectious.
  set incubation-length incubation-length + 1
  if incubation-length > incubation-time
  [
    set exposed? false
    set infectious? true
    set infectious-time random-gamma infectious-alpha infectious-lambda                ;; A newly infectious individual selects an infectious-time from the gamma-distribution and its infection-length is set to 0.
    set infectious-length 0
  ]
end

to chance-of-recovery
  set infectious-length infectious-length + 1
  if infectious-length > infectious-time                              ;; When an infectious individual lasted over its infection-time, the goose will die off or recover.
  [ifelse random-float 100 > mortality
    [set infectious? false
     set recovered? true]
    [die]
  ]
end

to compute-maximum-infectious                                     ;; A vector of the number of infectious individuals at each tick is stored. The maximum and time of the maximum are computed.
  set number-infectious-bird lput count turtles with [infectious?] number-infectious-bird
  set maximum-infectious max number-infectious-bird
```

```
  set tick-at-maximum-infectious position maximum-infectious number-infectious-bird
end

;;;;;;;;;;;;;;;;;;;;;;;;;;;;;;;;;;;;;;;;;;;;;;;;;;;;;;;;;;;;;;;;;;;;;;;;;;;;;;;;;;;;;;;;;;;;;;;;;;;;;;;;;;;;;;;;;;;;;;;;;;;;;;;;;;;;;;;;;;;;;;;;;;;;;;;;;;;;;;;;;;;;;;;;;;;;;;;;;;;;;;;;;;;;;;;;;;;;;;;;;;;;;;;;;;;;;;;;;;;;;

;;;;;  Flocking procedure  ;;;;;;;;;;;;;;;;;;;;;;;;;;;;;;;;;;;;;;;;;;;;;;;;;;;;;;;;;;;;;;;;;;;;;;;;;;;;;;;;;;;;;;;;;;;;;;;;;;;;;;;;;;;;;;;;;;;;;;;;;;;;;;;;;;;;;;;;;;;;;;;;;;;;;;;;;;;;;;;;;;;;;;;;;;;;;;;;;;;;;;;

;;;;;;;;;;;;;;;;;;;;;;;;;;;;;;;;;;;;;;;;;;;;;;;;;;;;;;;;;;;;;;;;;;;;;;;;;;;;;;;;;;;;;;;;;;;;;;;;;;;;;;;;;;;;;;;;;;;;;;;;;;;;;;;;;;;;;;;;;;;;;;;;;;;;;;;;;;;;;;;;;;;;;;;;;;;;;;;;;;;;;;;;;;;;;;;;;;;;;;;;;;;;;;;;;;;;;;;;;;;;;;

;;;;;  Wilensky, U. (1998). NetLogo Flocking model. http://ccl.northwestern.edu/netlogo/models/Flocking. Center for Connected Learning and Computer-Based Modeling, Northwestern University, Evanston, IL. ;;;;;;;;;;;;;;;;

;;;;;;;;;;;;;;;;;;;;;;;;;;;;;;;;;;;;;;;;;;;;;;;;;;;;;;;;;;;;;;;;;;;;;;;;;;;;;;;;;;;;;;;;;;;;;;;;;;;;;;;;;;;;;;;;;;;;;;;;;;;;;;;;;;;;;;;;;;;;;;;;;;;;;;;;;;;;;;;;;;;;;;;;;;;;;;;;;;;;;;;;;;;;;;;;;;;;;;;;;;;;;;;;;;;;;;;;;;;;;;


to flock
  let water gis:raster-sample site self
  find-flockmates
  if any? flockmates
    [ find-nearest-neighbor
      ifelse distance nearest-neighbor < 0.05                    ;; All flockmates try to maintain at least 5m of a basic social distance.
         [separate]
         [ align
         cohere ]]
   if count flockmates = 0
     [if water = 1
       [set heading random 360]]                          ;; Inside a wetland, birds take a random direction when no bird is around.
end

to find-flockmates
  set flockmates other turtles in-radius 10                    ;; Find other birds within 1.0km in radius.
end
```

```
to find-nearest-neighbor
  set nearest-neighbor min-one-of flockmates [distance myself]
end

to separate
  turn-away ([heading] of nearest-neighbor) random 30                    ;; Max-separate-turn is <30 degree.
 end

to align
  turn-towards average-flockmate-heading random 30                    ;; Max-align-turn is <30 degree.
end

to-report average-flockmate-heading                                    ;; Using trigonometry to compute average the
heading variables.
  let x-component sum [dx] of flockmates
  let y-component sum [dy] of flockmates
  ifelse x-component = 0 and y-component = 0
    [ report heading ]
    [ report atan x-component y-component ]
end

;;; COHERE

to cohere  ;; turtle procedure
  turn-towards average-heading-towards-flockmates random 30             ;; max-cohere-turn is <30 degree.
end

to-report average-heading-towards-flockmates
  let x-component mean [sin (towards myself + 180)] of flockmates        ;; The "towards myself" indicates
the heading from the other turtle to the focal agent. Add 180 degree to calculate the heading toward the
other turtle.
```

```
  let y-component mean [cos (towards myself + 180)] of flockmates
  ifelse x-component = 0 and y-component = 0
    [ report heading ]
    [ report atan x-component y-component ]
end

;;; HELPER PROCEDURES

to turn-towards [new-heading max-turn]
  turn-at-most (subtract-headings new-heading heading) max-turn
end


to turn-away [new-heading max-turn]
  turn-at-most (subtract-headings heading new-heading) max-turn
end


to turn-at-most [turn max-turn]
  ifelse abs turn > max-turn
    [ ifelse turn > 0
        [ rt max-turn ]
        [ lt max-turn ] ]                                   ;; Turn right by "turn" degrees or left if "turn" is negative, but never turn more than "max-turn" degrees
    [ rt turn ]
end


;;;;;;;;;;;;;;;;;;;;;;;;;;;;;;;;;;;;;;;;;;;;;;;;;;;;;;;;;;;;;;;;;;;;;;;;;;;;;;;;;;;;;;;;;;;;;;;;;;;;;;;;;;;;;;;;;;;;;;;;;;;;;;;;;;;;;;;;;;;;
;;;;;  Movements of geese: Stage 1 ;;;;;;;;;;;;;;;;;;;;;;;;;;;;;;;;;;;;;;;;;;;;;;;;;;;;;;;;;;;;;;;;;;;;;;;;;;;;;;;;;;;;;;;;;;;;;;;;;;;;;;;
;;;;;;;;;;;;;;;;;;;;;;;;;;;;;;;;;;;;;;;;;;;;;;;;;;;;;;;;;;;;;;;;;;;;;;;;;;;;;;;;;;;;;;;;;;;;;;;;;;;;;;;;;;;;;;;;;;;;;;;;;;;;;;;;;;;;;;;;;;;;

to molt                                                ;; Movements of geese before 45.7 days (1096.8 hours or ticks) in molting wetlands only
   let water gis:raster-sample site self
```

```
  ifelse water = 1
      [ set stationed? True
        ifelse random-float 100 < (100 - wetland-changeI / ticks-per-day)          ;; The chance of inter-
wetland movements in the second stage (converted by ticks)
          [forward random-exponential 2.0]                                        ;; Movements when turtles are in
wetlands with exponential distribution of houly displacements (mean of 200 m/hr) based on telemetry
data
          [forward random-exponential 174]]                                       ;; Occasional interwetland
movements in the second stage (mean of 17.4 km/hr) ==> No telemetry data, so use the stage 2 info.
      [heading-for-molt]
end



;;;;;;;;;;;;;;;;;;;;;;;;;;;;;;;;;;;;;;;;;;;;;;;;;;;;;;;;;;;;;;;;;;;;;;;;;;;;;;;;;;;;;;;;;;;;;;;;;;;;;;;;;;;;;;;;;;;;;;;;;;;;;;;;;;;;;;;;;;;;;;;;;;;;;;;;;;;;;;;;;;;;
;;;;;  Movements of geese: Stage 2 ;;;;;;;;;;;;;;;;;;;;;;;;;;;;;;;;;;;;;;;;;;;;;;;;;;;;;;;;;;;;;;;;;;;;;;;;;;;;;;;;;;;;;;;;;;;;;;;;;;;;;;;;;;;;;;;;;;;;;;;;;
;;;;;;;;;;;;;;;;;;;;;;;;;;;;;;;;;;;;;;;;;;;;;;;;;;;;;;;;;;;;;;;;;;;;;;;;;;;;;;;;;;;;;;;;;;;;;;;;;;;;;;;;;;;;;;;;;;;;;;;;;;;;;;;;;;;;;;;;;;;;;;;;;;;;;;;;;;;;;;;;;;;;;;

to move

  let water gis:raster-sample site self
  ifelse water = 1
      [ set stationed? True                                       ;; To count the number of agents in wetlands
        ifelse random-float 100 < (100 - wetland-changeII / ticks-per-day)          ;; The chance of inter-
wetland movements in the second stage (converted by ticks)
          [forward random-exponential 2.3]                                        ;; Movements when turtles are in
wetlands with exponential distribution of houly displacements (mean of 230 m/hr) based on telemetry
data
          [forward random-exponential 174]]                                       ;; Occasional interwetland
movements in the second stage (mean of 17.4 km/hr) based on telemetry data
      [heading-for-wetland]
end



;;;;;;;;;;;;;;;;;;;;;;;;;;;;;;;;;;;;;;;;;;;;;;;;;;;;;;;;;;;;;;;;;;;;;;;;;;;;;;;;;;;;;;;;;;;;;;;;;;;;;;;;;;;;;;;;;;;;;;;;;;;;;;;;;;;;;;;;;;;;;;;;;;;;;;;;;;;;;;;;;;;;;
;;;;;  Movements of geese: Stage 3 ;;;;;;;;;;;;;;;;;;;;;;;;;;;;;;;;;;;;;;;;;;;;;;;;;;;;;;;;;;;;;;;;;;;;;;;;;;;;;;;;;;;;;;;;;;;;;;;;;;;;;;;;;;;;;;;;;;;;;;;;;
```

```
;;;;;;;;;;;;;;;;;;;;;;;;;;;;;;;;;;;;;;;;;;;;;;;;;;;;;;;;;;;;;;;;;;;;;;;;;;;;;;;;;;;;;;;;;;;;;;;;;;;;;;;;;;;;;;;;;;;;;;;;;;;;;;;;;;

to premigrate

  let water gis:raster-sample site self

  ifelse water = 1
    [ set stationed? True                                    ;; To count the number of agents in wetlands
      ifelse random-float 100 < (100 - wetland-changeIII / ticks-per-day)           ;; The chance of inter-wetland movements in the third stage (converted by ticks)
        [forward random-exponential 4.1]                                  ;; Movements when turtles are in wetlands with exponential distribution of houly displacements (mean of 410 m/hr) based on telemetry data
        [forward random-exponential 193]]                                  ;; Occasional interwetland movements in the third stage (mean of 19.3 km/hr) based on telemetry data
    [heading-for-wetland]
end

;;;;;;;;;;;;;;;;;;;;;;;;;;;;;;;;;;;;;;;;;;;;;;;;;;;;;;;;;;;;;;;;;;;;;;;;;;;;;;;;;;;;;;;;;;;;;;;;;;;;;;;;;;;;;;;;;;;;;;;;;;;;;;;;;;
;;;;;  Moving to molting wetlands: Stage 1 ;;;;;;;;;;;;;;;;;;;;;;;;;;;;;;;;;;;;;;;;;;;;;;;;;;;;;;;;;;;;;;;;;;;;;;;;;;;;;;;;
;;;;;;;;;;;;;;;;;;;;;;;;;;;;;;;;;;;;;;;;;;;;;;;;;;;;;;;;;;;;;;;;;;;;;;;;;;;;;;;;;;;;;;;;;;;;;;;;;;;;;;;;;;;;;;;;;;;;;;;;;;;;;;;;;;

to heading-for-molt
                                                          ;; If agnets are outside any major wetlands, perform the molting migrations to the nearest one of major wetlands
   let x xcor
   let y ycor

   let Moltattract_Here gis:raster-sample moltattract self

   ifelse ((Moltattract_Here <= 0) or (Moltattract_Here >= 0))                    ;; Check for Nodata
   [
     let direction 0
     let min_melevation Moltattract_Here
```

```
   if ((x - 1) >= 0)                                   ;; Determine a direction along the distance
gradient (west)
   [
     let coor list ( x - 1 )  ( y )
     let MElevation_Left gis:raster-sample moltattract coor
     if (MElevation_Left < min_melevation)
     [
       set direction "WW"
       set min_melevation MElevation_Left
     ]
   ]

   if ((x - 1) >= 0) and ((y - 1) > 0)                 ;; Determine a direction along the
distance gradient (south-west)
   [
     let coor list ( x - 1 )  (y - 1)
     let MElevation_Left gis:raster-sample moltattract coor
     if (MElevation_Left < min_melevation)
     [
       set direction "SW"
       set min_melevation MElevation_Left
     ]
   ]


   if ((y - 1) > 0)                                    ;; Determine a direction along the distance
gradient (south)
   [
     let coor list ( x )  ( y - 1 )
     let MElevation_Top gis:raster-sample moltattract coor
     if (MElevation_Top < min_melevation)
     [
```

```
      set direction "SS"
      set min_melevation MElevation_Top
    ]
  ]

  if ((x + 1) < width )  and ((y - 1) > 0)                    ;; Determine a direction along the distance gradient (south-east)
  [
    let coor list ( x + 1 ) ( y - 1 )
     let MElevation_Right gis:raster-sample moltattract coor
    if (MElevation_Right < min_melevation)
    [
      set direction "SE"
      set min_melevation MElevation_Right
    ]
  ]


  if ((x + 1) < width )                                       ;; Determine a direction along the distance gradient (east)
  [
    let coor list ( x + 1 )  ( y )
     let MElevation_Right gis:raster-sample moltattract coor
    if (MElevation_Right < min_melevation)
    [
      set direction "EE"
      set min_melevation MElevation_Right
    ]
  ]

  if ((x + 1) < width ) and ((y + 1) < height)                ;; Determine a direction along the distance gradient (north-east)
  [
    let coor list ( x + 1 )  ( y + 1 )
```

```
 let MElevation_Right gis:raster-sample moltattract coor
 if (MElevation_Right < min_melevation)
 [
   set direction "NE"
   set min_melevation MElevation_Right
 ]
]

if ((y + 1) < height)                                   ;; Determine a direction along the distance
gradient (north)
[
 let coor list  ( x )  ( y + 1 )
 let MElevation_Down gis:raster-sample moltattract coor ;;
 if (MElevation_Down < min_melevation)
 [
   set direction "NN"
   set min_melevation MElevation_Down
 ]
]

if ((x - 1) >= 0) and ((y + 1) < height)                ;; Determine a direction along the
distance gradient (north-west)
[
 let coor list ( x - 1 )  ( y + 1 )
  let MElevation_Right gis:raster-sample moltattract coor ;;
 if (MElevation_Right < min_melevation)
 [
   set direction "NW"
   set min_melevation MElevation_Right
 ]
]

if (direction = "WW" ) [ set heading random-normal 270 15]
```

```
      if (direction = "SW" ) [ set heading random-normal 225 15]
      if (direction = "SS" ) [ set heading random-normal 180 15]
      if (direction = "SE" ) [ set heading random-normal 135 15]
      if (direction = "EE" ) [ set heading random-normal 90 15]
      if (direction = "NE" ) [ set heading  random-normal 45 15]
      if (direction = "NN" ) [ set heading random-normal 0 15]
      if (direction = "NW" ) [ set heading random-normal 315 15]

    flight]
    [set heading random 360 forward random 100]                              ;; In case of Nodata


end


;;;;;;;;;;;;;;;;;;;;;;;;;;;;;;;;;;;;;;;;;;;;;;;;;;;;;;;;;;;;;;;;;;;;;;;;;;;;;;;;;;;;;;;;;;;;;;;;;;;;;;;;;;;;;;;;;;;;;;;;;;;;;;;;;;;;;;;;;;;;;;;;;;;;;;;;;;;;;;;;;;;;;;;;;;;;;;;;;;;;
;;;;;;;;;;;;;;;;;;
;;;;;;  Moving to available wetlands: Stage 2-3
;;;;;;;;;;;;;;;;;;;;;;;;;;;;;;;;;;;;;;;;;;;;;;;;;;;;;;;;;;;;;;;;;;;;;;;;;;;;;;;;;;;;;;;;;;;;;;;;;;;;;;;;;;;;;;;;;;;;;;;;;;;;;;;;;;;;;;;
;;;;;;;;;;;;;;;;;;;;;;;;;;;;;;;;;;;;;;;;;;;;;;;;;;;;;;;;;;;;;;;;;;;;;;;;;;;;;;;;;;;;;;;;;;;;;;;;;;;;;;;;;;;;;;;;;;;;;;;;;;;;;;;;;;;;;;;;;;;;;;;;;;;;;;;;;;;;;;;;;;;;;;;;;;;;;;;;;;;
;;;;;;;;;;;;;;;;;;


to heading-for-wetland
                                                        ;; Movements when turtles are out of wetlands
(heading toward wetlands)
    let x xcor
    let y ycor

    let Elevation_Here gis:raster-sample elevation self

    ifelse ((Elevation_Here <= 0) or (Elevation_Here >= 0))                ;; Check for Nodata
    [
      let direction 0
      let min_elevation Elevation_Here
```

```
if ((x - 1) > 0)
[
  let coor list ( x - 1 )  ( y )
  let Elevation_Left gis:raster-sample elevation coor
  if (Elevation_Left < min_elevation)
  [
    set direction "WW"
    set min_elevation Elevation_Left
  ]
]

if ((x - 1) > 0) and ((y - 1) > 0)
[
  let coor list ( x - 1 )  (y - 1)
  let Elevation_Left gis:raster-sample elevation coor
  if (Elevation_Left < min_elevation)
  [
    set direction "SW"
    set min_elevation Elevation_Left
  ]
]

if ((y - 1) > 0)
[
  let coor list ( x )  ( y - 1 )
  let Elevation_Top gis:raster-sample elevation coor
  if (Elevation_Top < min_elevation)
  [
    set direction "SS"
    set min_elevation Elevation_Top
  ]
]
```

```
if ((x + 1) < width )  and ((y - 1) > 0)
[
  let coor list ( x + 1 ) ( y - 1 )
   let Elevation_Right gis:raster-sample elevation coor
  if (Elevation_Right < min_elevation)
  [
    set direction "SE"
    set min_elevation Elevation_Right
  ]
]


if ((x + 1) < width )
[
  let coor list ( x + 1 )  ( y )
   let Elevation_Right gis:raster-sample elevation coor
  if (Elevation_Right < min_elevation)
  [
    set direction "EE"
    set min_elevation Elevation_Right
  ]
]

if ((x + 1) < width ) and ((y + 1) < height)
[
  let coor list ( x + 1 )  ( y + 1 )
   let Elevation_Right gis:raster-sample elevation coor
  if (Elevation_Right < min_elevation)
  [
    set direction "NE"
    set min_elevation Elevation_Right
  ]
```

```
]

if ((y + 1) < height)
[
  let coor list  ( x )  ( y + 1 )
  let Elevation_Down gis:raster-sample elevation coor
  if (Elevation_Down < min_elevation)
  [
    set direction "NN"
    set min_elevation Elevation_Down
  ]
]

if ((x - 1) > 0) and ((y + 1) < height)
[
  let coor list ( x - 1 )  ( y + 1 )
   let Elevation_Right gis:raster-sample elevation coor
  if (Elevation_Right < min_elevation)
  [
    set direction "NW"
    set min_elevation Elevation_Right
  ]
]

if (direction = "WW" ) [ set heading random-normal 270 15]
if (direction = "SW" ) [ set heading random-normal 225 15]
if (direction = "SS" ) [ set heading random-normal 180 15]
if (direction = "SE" ) [ set heading random-normal 135 15]
if (direction = "EE" ) [ set heading random-normal 90 15]
if (direction = "NE" ) [ set heading  random-normal 45 15]
if (direction = "NN" ) [ set heading random-normal 0 15]
if (direction = "NW" ) [ set heading random-normal 315 15]
```

```
   flight]
   [set heading random 360 forward random 100]                          ;; In case of Nodata

end

;;;;;;;;;;;;;;;;;;;;;;;;;;;;;;;;;;;;;;;;;;;;;;;;;;;;;;;;;;;
;;;;;;;;; Flight between wetlands ;;;;;;;;;;;;;;;;
;;;;;;;;;;;;;;;;;;;;;;;;;;;;;;;;;;;;;;;;;;;;;;;;;;;;;;;;;;;

to flight

  if ticks <= 24 * 5                                  ;; Initial distribution allowing the
asynchlonouse arrival to molting wetlands for 5 days
   [forward random 10]

  if ticks > 24 * 5 and ticks < 1097                        ;; Interwetland movements during the
stage 1 (molting stage) that uses molting lakes only
   [ let wdist gis:raster-sample moltattract self
     if (wdist >= 2000) [forward wdist / 100]
     if (wdist < 2000) [forward random 10]
   ]

  if ticks >= 1097                                    ;; Interwetland movements during the stage
2-3 that uses all types of wetlands
   [ let awdist gis:raster-sample elevation self
     if (awdist >= 2000) [forward awdist / 100]
     if (awdist < 2000) [forward random 10]
   ]

end
@#$#@#$#@
```
